# Outcomes of Anastrozole, Letrozole, and Exemestane in Patients With Postmenopausal Breast Cancer

**DOI:** 10.1001/jamanetworkopen.2025.50842

**Published:** 2025-12-26

**Authors:** Elise Dumas, Anne-Sophie Hamy, Kerollos Nashat Wanis, Floriane Jochum, Florence Coussy, Sylvie Giacchetti, Thomas Gaillard, Enora Laas, Sophie Houzard, Christine Le Bihan-Benjamin, Fabien Reyal, Paul Gougis, Mats Julius Stensrud

**Affiliations:** 1 Institute of Mathematics, École Polytechnique Fédérale de Lausanne, Lausanne, Switzerland; 2 Residual Tumor and Response to Treatment Laboratory, RT2Lab, Translational Research Department, Institut National de la Santé et de la Recherche Médicale (INSERM), U932 Immunity and Cancer, Université Paris Cité, Paris, France; 3 Department of Medical Oncology, Institut Curie, Université Paris Cité, Paris, France; 4 Departments of Breast Surgical Oncology and Health Services Research and Institute for Data Science in Oncology, The University of Texas MD Anderson Cancer Center, Houston; 5 Department of Gynecology, Strasbourg University Hospital, Strasbourg, France; 6 Sénopole, Hôpital Saint-Louis, Assistance Publique–Hôpitaux de Paris (AP-HP), Université Paris Cité, Paris, France; 7 Department of Surgical Oncology, Université Paris Cité, Institut Curie, Paris, France; 8 Health Data and Assessment, Health Survey Data Science and Assessment Division, Institut National du Cancer (INCa), Boulogne-Billancourt, France; 9 Department of Surgery, Institut Jean Godinot, Reims, France; 10 Department of Medical Oncology, Pitié-Salpêtrière Hospital, Sorbonne Université, Institut Universitaire du Cancer, Paris, France; 11 Clinical Investigation Center (CIC-1901), INSERM, Department of Pharmacology, Pitié-Salpêtrière Hospital, Sorbonne Université, Paris, France

## Abstract

**Importance:**

Third-generation aromatase inhibitors (AIs)—anastrozole, letrozole, and exemestane—are standard adjuvant endocrine therapy for women with postmenopausal hormone receptor (HR)–positive early-stage breast cancer. However, little is known about their comparative effectiveness in clinical settings.

**Objective:**

To compare adjuvant anastrozole, letrozole, and exemestane in terms of disease-free survival (DFS) and overall survival (OS) in women with postmenopausal HR-positive early-stage breast cancer.

**Design, Setting, and Participants:**

This comparative effectiveness study conducted in France emulated a target trial using national medicoadministrative data. The patient cohort was derived from the updated French Early Breast Cancer Cohort and consisted of women aged 50 to 75 years with early-stage breast cancer who were diagnosed between January 1, 2011, and December 31, 2020, and followed up through December 31, 2021. All women initiated adjuvant AI therapy without ovarian suppression. Data analysis was performed from November 2024 to May 2025.

**Exposures:**

Upfront adjuvant anastrozole, letrozole, and exemestane, evaluated under natural persistence and under a hypothetical intervention ensuring perfect persistence for 5 years.

**Main Outcomes and Measures:**

The primary end points were DFS and OS. Both DFS and OS were estimated using adjusted Kaplan-Meier curves.

**Results:**

Among 148 436 women (median [IQR] age, 64 [59-69] years) included in the analyses, 38.5% initiated anastrozole, 52.9% initiated letrozole, and 8.5% initiated exemestane. After a median (IQR) follow-up of 63 (34-94) months, the 8-year DFS under natural persistence was estimated to be lower for the exemestane group (79.1%; 95% CI, 78.1%-80.0%) compared with the anastrozole (81.0%; 95% CI, 80.6%-81.5%) and letrozole (81.1%; 95% CI, 80.7%-81.5%) groups. Similarly, the 8-year OS was 88.8% (95% CI, 88.0%-89.6%) for the exemestane group compared with 90.5% (95% CI, 90.2%-90.8%) for the anastrozole group and 89.9% (95% CI, 89.6%-90.2%) for the letrozole group. Patients who initiated exemestane were also more likely to discontinue treatment within 5 years of therapy than their counterparts taking the other AIs (39.3% [95% CI, 38.3%-40.3%] in the exemestane group vs 35.1% [95% CI, 34.7%-35.6%] in the anastrozole group and 35.0% [95% CI, 34.6%-35.4%] in the letrozole group). However, the lower DFS and OS observed for exemestane compared with letrozole and anastrozole persisted under perfect persistence.

**Conclusions and Relevance:**

The findings of this comparative effectiveness study using a target trial emulation framework suggest that adjuvant endocrine therapy with exemestane may result in slightly lower DFS and OS compared with anastrozole and letrozole in patients with postmenopausal HR-positive early-stage breast cancer. The findings favor anastrozole or letrozole as initial treatment and highlight the need for future studies to inform AI selection.

## Key Points

**Question:**

Are there differences in the clinical outcomes associated with anastrozole, letrozole, and exemestane as adjuvant endocrine therapy in women with postmenopausal hormone receptor–positive early-stage breast cancer?

**Findings:**

In this comparative effectiveness study emulating a target trial and including 148 436 postmenopausal women in France, exemestane was associated with lower disease-free survival and overall survival after 8 years compared with anastrozole and letrozole, both under natural and perfect persistence.

**Meaning:**

These findings suggest higher risks of recurrence and death with exemestane and thus may favor anastrozole and letrozole as initial treatment; future studies may inform aromatase inhibitor molecule selection in clinical practice.

## Teaser

This comparative effectiveness research compares disease-free survival and overall survival 5 and 8 years after the initiation of endocrine therapy in postmenopausal women with hormone receptor–positive early-stage breast cancer.

## Introduction

Breast cancer (BC) is the most commonly diagnosed cancer among women and the leading cause of cancer death in women worldwide.^1^ Approximately 70% to 80% of breast tumors express hormone receptors (HRs; HR-positive tumors).^2^ Five to 10 years of daily oral endocrine therapy are recommended to prevent disease recurrence in patients diagnosed with an early-stage HR-positive tumor.^3^ Until the 2000s, tamoxifen, a selective estrogen receptor modulator, was the standard adjuvant endocrine therapy for patients with postmenopausal early-stage HR-positive BC.^4^ Third-generation aromatase inhibitors (AIs)—specifically, anastrozole, letrozole, and exemestane—have emerged more recently.^5^ AIs reduce the production of estrogen in plasma and tissues by inhibiting the conversion of androgens to estrogens operated by the aromatase enzyme.^6^ The 3 AIs, used either upfront for 5 years^7,8^ or sequentially after 2 to 3 years of tamoxifen,^9^ have been shown to be more effective than tamoxifen alone in reducing the risk of recurrence and BC-specific mortality in postmenopausal women with HR-positive early-stage BC and have become standard of care in this setting.^10,11^

The 3 AIs have different pharmacological properties.^12^ Steroidal exemestane irreversibly binds to the aromatase enzyme, whereas the nonsteroidal anastrozole and letrozole are reversible competitive inhibitors of aromatase.^13^ There is evidence that the 3 drugs do not have the same potency for serum estrogen suppression.^14,15,16^ They might also exhibit different toxicity profiles; for example, exemestane may cause fewer skeletal and lipid-related adverse effects.^17,18,19,20^ Three randomized clinical trials (RCTs) conducted head-to-head comparisons of adjuvant AIs in postmenopausal women: the FATA-GIM3 trial compared the 3 AIs with each other,^21^ the MA.27 trial compared exemestane with letrozole,^22^ and the FACE trial (Randomized Phase III Femara Versus Anastrozole Clinical Evaluation)^23^ compared anastrozole with letrozole in patients with node-positive disease only. None of these 3 trials found strong evidence of differences in recurrence risks among the AIs. Therefore, international guidelines do not recommend one drug over the other, and the choice of AI is largely determined by prescription habits.^10,24^

RCTs are the gold standard for establishing causal effects.^25^ However, RCTs may be limited by insufficient power to detect small effect sizes.^26,27^ The FATA-GIM3 trial, for example, was initially designed to detect a 2% difference after 5 years; but the trial was ultimately underpowered due to a lower-than-expected number of events. In addition, patient persistence to prescribed therapy may be higher in RCTs than in clinical settings.^28^ Thus, the outcomes of the 3 AIs might differ, even if the previous RCTs did not report significant results. This setting motivated us to conduct a study comparing adjuvant anastrozole, letrozole, and exemestane in terms of disease-free survival (DFS) and overall survival (OS) in women with postmenopausal HR-positive early-stage BC. We derived a cohort of these patients from a nationwide French medicoadministrative dataset.

## Methods

The French data protection agency Commission Nationale de l'Informatique et des Libertés authorized this comparative effectiveness study. In accordance with French regulations applicable to Système National des Données de Santé (SNDS) data, no informed consent was required because the data used in the study were deidentified and reused for research purposes. We followed the International Society for Pharmacoeconomics and Outcomes Research (ISPOR)^29^ reporting guideline.

### Data Source and Cohort Inclusion and Exclusion Criteria

**Figure 1.**
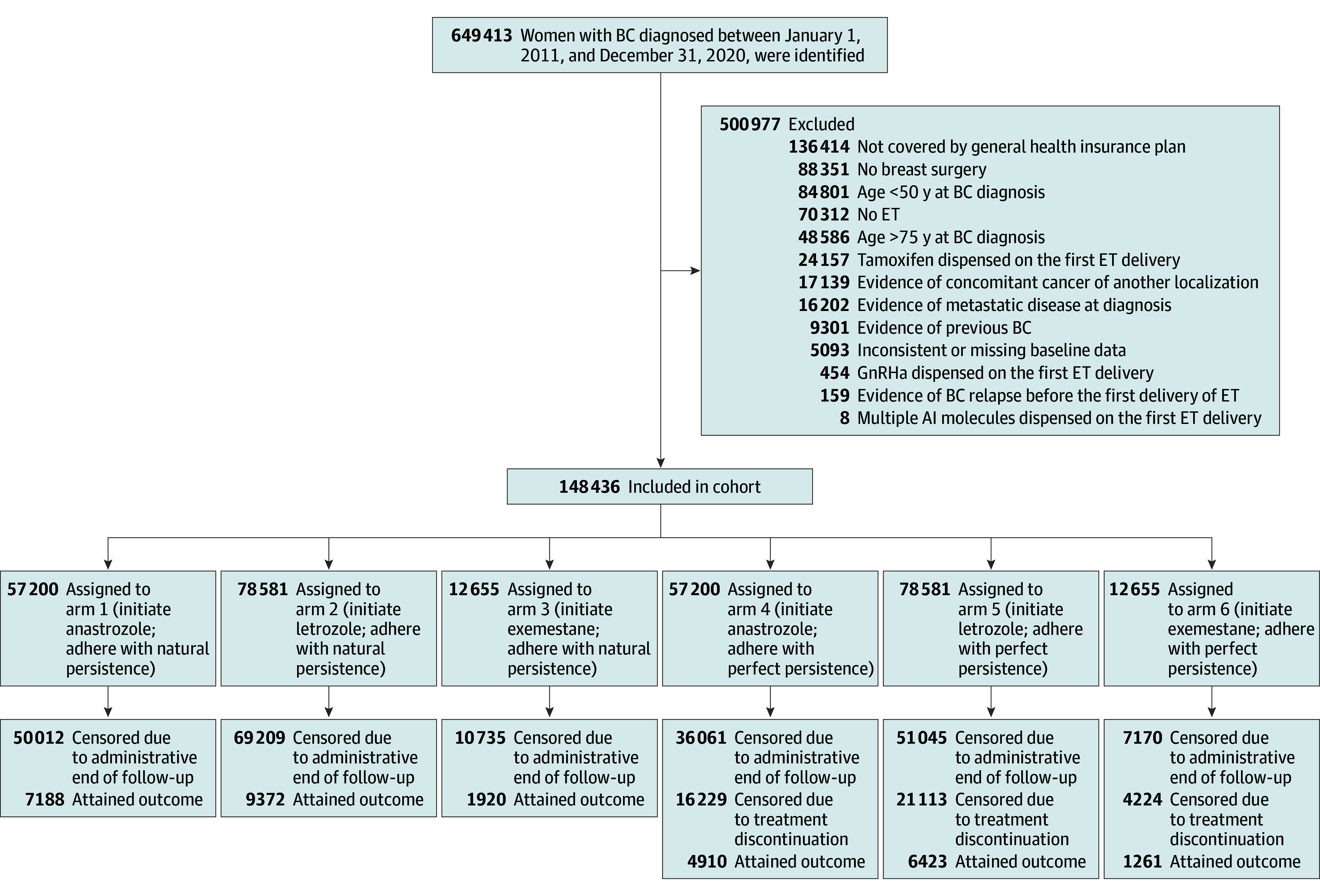
Study Flow Diagram The final row shows the number of patients who attained the DFS outcome. Data for patients artificially assigned to arms 1 and 4, 2 and 5, and 3 and 6 are exact copies of the data for patients who initiated anastrozole, letrozole, and exemestane, respectively. AI indicates aromatase inhibitor; BC, breast cancer; ET, endocrine therapy; GnRHa, gonadotropin-releasing hormone analogue.

We used nationwide retrospective data from the French Early Breast Cancer Cohort (FRESH) cohort,^30^ updated in 2024 and released from the SNDS database within the Oncology Data Platform at France’s Institut National du Cancer.^31,32^ The updated FRESH cohort includes women 18 years or older with early-stage BC who were newly diagnosed between January 1, 2011, and December 31, 2020. The women were identified by a diagnosis code for BC within the period considered. The FRESH cohort excludes individuals not covered by the main health insurance scheme (Régime Général); not undergoing breast surgery; with evidence of concomitant cancer of another localization, previous cancer, or metastatic disease at diagnosis; or with inconsistent or missing baseline data. In this study, we further excluded patients diagnosed before age 50 years or after age 75 years, not initiating endocrine therapy within 1 year after surgery, initiating tamoxifen or AI with concurrent ovarian suppression, or with evidence of disease recurrence before endocrine therapy onset (Figure 1). The eMethods in Supplement 1 provides details on the French health care insurance and the cohort’s age restriction.

### Endocrine Therapy Uptake and Persistence

The uptake of endocrine therapy was determined from the outpatient dispensing of anastrozole, letrozole, or exemestane at initiation and during follow-up and tamoxifen during follow-up. Endocrine therapy persistence was time varying, updated every month, and defined as no period of endocrine therapy discontinuation that lasted more than 30 consecutive days. We considered a patient to have discontinued endocrine therapy for 30 consecutive days if the patient had not been dispensed an AI or tamoxifen in the previous 30 consecutive days and could not be covered, in the previous 30 consecutive days, by an excess of AI or tamoxifen from a previous dispensing. Details are available in the eMethods in Supplement 1.

### Treatment Strategies

We emulated a target trial using observational data.^33,34,35^ In the hypothetical target trial, patients would be randomly assigned to 6 arms defined by different AI molecules at initiation and different persistence patterns. Specifically, patients assigned to arms 1 to 3 would be asked to initiate anastrozole (arm 1), letrozole (arm 2), or exemestane (arm 3) and would then be allowed to adhere to treatment as they naturally would (natural persistence). Patients assigned to arms 4 to 6 would be asked to initiate anastrozole (arm 4), letrozole (arm 5), or exemestane (arm 6) and would then adhere to treatment continuously for at least 5 years or until BC recurrence (perfect persistence). In all 6 arms, patients would be allowed to switch treatment to tamoxifen or to another AI.

### Outcomes

The primary end points were DFS and OS. We defined DFS as the absence of BC recurrence (including locoregional, contralateral, or distant recurrence) and death from any cause. The identification of BC recurrence was based on procedures, molecules, or diagnoses indicative of cancer recurrence or metastasis (eMethods in Supplement 1). We defined OS as the absence of death from any cause. We set time 0 as the date of the first adjuvant AI delivery.

Secondary end points were time to first endocrine therapy discontinuation and the cumulative distribution of incident adverse events. Adverse events were identified through hospitalization diagnosis codes, procedures, medical devices used, and treatments received. Adverse events included dyslipidemia, diabetes, cardiovascular events, osteoporosis, bone fracture, depression or anxiety, gastrointestinal adverse effects, and dermatologic adverse effects (eMethods and eTable 1 in Supplement 1).

### Statistical Analysis

In the emulation of treatment arms 1 to 3 (natural persistence), we reweighted the population using inverse probability of treatment weighting (IPTW) to account for confounding bias due to the choice of the AI molecule at baseline. IPTW was based on confounding factors at baseline: age, deprivation index, number and type of comorbid conditions, nodal status, *ERBB2* (formerly *HER2*) status, mammographic screening in the year preceding BC diagnosis, type of breast surgery, type of medical structure, radiotherapy, chemotherapy, and chemotherapy setting.

In the emulation of treatment arms 4 to 6 (perfect persistence), we censored patients at first endocrine therapy discontinuation and reweighted the population using both IPTW for confounding bias due to the choice of the molecule at baseline and inverse probability of censoring weights (IPCW) for selection bias due to censoring at endocrine therapy discontinuation. IPCW calculations were updated every month and were based on both the baseline and time-varying confounding factors. The time-varying covariates included the occurrence of an adverse event, the delivery of antipain or anti-inflammatory drugs, and the delivery of vitamin D or calcium supplementation.

We estimated DFS and OS on the marginal population from weighted Kaplan-Meier survival curves. We reported DFS and OS proportions and their associated 95% CIs at 5 and 8 years after initiating AI treatment. We used the nonparametric bootstrap with 1000 iterations to estimate 95% CIs based on percentiles. The 5-year DFS estimates were compared with findings from the FATA-GIM3,^21^ MA.27,^22^ and FACE^23^ trials. We also estimated the cumulative incidence of first endocrine therapy discontinuation and incident adverse events up to 5 years under observed persistence (arms 1-3) on the weighted population. Adverse event analyses were restricted to patients without a history of the respective event in the year preceding endocrine therapy initiation. Additionally, for descriptive purposes only, we presented the distribution of endocrine therapy molecule switches across the unweighted population.

We performed sensitivity analyses consisting of the emulation of alternative target trials. We emulated a target trial that included patients treated with chemotherapy only. The eMethods in Supplement 1 describes the rationale and implementation of the sensitivity analyses.

Data analysis was performed from November 2024 to May 2025. R, version 3.6.3 (R Project for Statistical Computing), was used in the analysis.

## Results

### Patients and Events

We included 148 436 women (median [IQR] age, 64 [59-69] years) in the study (Figure 1, Table 1). Of these patients, 38.5% initiated anastrozole, 52.9% initiated letrozole, and 8.5% initiated exemestane at the endocrine therapy onset. At a median (IQR) follow-up of 63 (34-94) months, there were 18 480 DFS events (12.4%) and 9449 OS events (6.4%).

**Table 1.** Patient Baseline Characteristics by Aromatase Inhibitor Molecule at Endocrine Therapy Initiation

Characteristic	Patients, No. (%)			
	Overall	Anastrozole	Letrozole	Exemestane
Total No.	148 436 (100)	57 200 (38.5)	78 581 (52.9)	12 655 (8.5)
Socioeconomic factors				
Age, median (IQR), y	64 (59-69)	64 (59-69)	64 (59-69)	64 (59-70)
Deprivation index				
First quintile (least deprived)	26 835 (18.1)	9770 (17.1)	14 712 (18.7)	2353 (18.6)
Second quintile	28 877 (19.5)	10 659 (18.6)	15 596 (19.8)	2622 (20.7)
Third quintile	29 389 (19.8)	11 408 (19.9)	15 382 (19.6)	2599 (20.5)
Fourth quintile	30 133 (20.3)	11 489 (20.1)	16 090 (20.5)	2554 (20.2)
Fifth quintile (most deprived)	30 909 (20.8)	12 904 (22.6)	15 541 (19.8)	2464 (19.5)
French overseas departments	2293 (1.5)	970 (1.7)	1260 (1.6)	63 (0.5)
No. of comorbid conditions				
0	48 572 (32.7)	17 860 (31.2)	26 697 (34.0)	4015 (31.7)
1	38 969 (26.3)	15 128 (26.4)	20 582 (26.2)	3259 (25.8)
2-4	53 337 (35.9)	21 111 (36.9)	27 564 (35.1)	4662 (36.8)
≥5	7558 (5.1)	3101 (5.4)	3738 (4.8)	719 (5.7)
Type of comorbid conditions^a^				
Endocrine and metabolism	51 661 (34.8)	20 528 (35.9)	26 591 (33.8)	4542 (35.9)
Cardiovascular	63 821 (43.0)	25 160 (44.0)	33 088 (42.1)	5573 (44.0)
Psychiatric	39 964 (26.9)	16 025 (28.0)	20 422 (26.0)	3517 (27.8)
Any other	26 556 (17.9)	10 537 (18.4)	13 565 (17.3)	2454 (19.4)
BC biological process				
Nodal status				
Node-negative	119 339 (80.4)	46 524 (81.3)	62 321 (79.3)	10 494 (82.9)
Node-positive	29 097 (19.6)	10 676 (18.7)	16 260 (20.7)	2161 (17.1)
*ERBB2* status				
Negative	137 996 (93.0)	53 338 (93.2)	72 744 (92.6)	11 914 (94.1)
Positive	10 440 (7.0)	3862 (6.8)	5837 (7.4)	741 (5.9)
BC diagnostic and treatments				
Mammographic screening before BC diagnosis				
No	58 000 (39.1)	22 002 (38.5)	30 965 (39.4)	5033 (39.8)
Yes	90 436 (60.9)	35 198 (61.5)	47 616 (60.6)	7622 (60.2)
Type of BC surgery				
Lumpectomy	118 390 (79.8)	46 374 (81.1)	61 851 (78.7)	10 165 (80.3)
Mastectomy	30 046 (20.2)	10 826 (18.9)	16 730 (21.3)	2490 (19.7)
Type of medical structure for BC surgery				
Comprehensive cancer centers	37 854 (25.5)	13 996 (24.5)	21 614 (27.5)	2244 (17.7)
Public hospitals	7780 (5.2)	3113 (5.4)	4062 (5.2)	605 (4.8)
For-profit private hospitals	60 635 (40.8)	23 920 (41.8)	30 131 (38.3)	6584 (52.0)
Nonprofit private hospitals	42 167 (28.4)	16 171 (28.3)	22 774 (29.0)	3222 (25.5)
Radiotherapy				
No	11 439 (7.7)	4110 (7.2)	6256 (8.0)	1073 (8.5)
Yes	136 997 (92.3)	53 090 (92.8)	72 325 (92.0)	11582 (91.5)
Chemotherapy				
No	94 791 (63.9)	37 052 (64.8)	48 966 (62.3)	8773 (69.3)
Yes	53 645 (36.1)	20 148 (35.2)	29 615 (37.7)	3882 (30.7)
Chemotherapy setting^b^				
Adjuvant only	46 053 (85.8)	17 448 (86.6)	25 285 (85.4)	3320 (85.5)
Neoadjuvant with or without adjuvant	7592 (14.2)	2700 (13.4)	4330 (14.6)	562 (14.5)

Abbreviation: BC, breast cancer.

^a^ Categories are not mutually exclusive.

^b^ Chemotherapy setting category is presented only for the subset of patients who received chemotherapy.

The median (IQR) age at BC diagnosis was 64 (59-69) years for patients who initiated anastrozole and letrozole and 64 (59-70) years for patients who initiated exemestane (Table 1). Overall, 7.0% of patients were diagnosed with *ERBB2*-positive tumors, and 19.6% had lymph node involvement; these percentages were similar across AI molecules. Fifty-two percent of patients who initiated exemestane were treated in for-profit private hospitals compared with 41.8% who received anastrozole and 38.3% who received letrozole. The proportion of patients who underwent chemotherapy was 30.7% in the exemestane group, 35.2% in the anastrozole group, and 37.7% in the letrozole group.

### DFS and OS Under Natural Persistence

**Figure 2.**
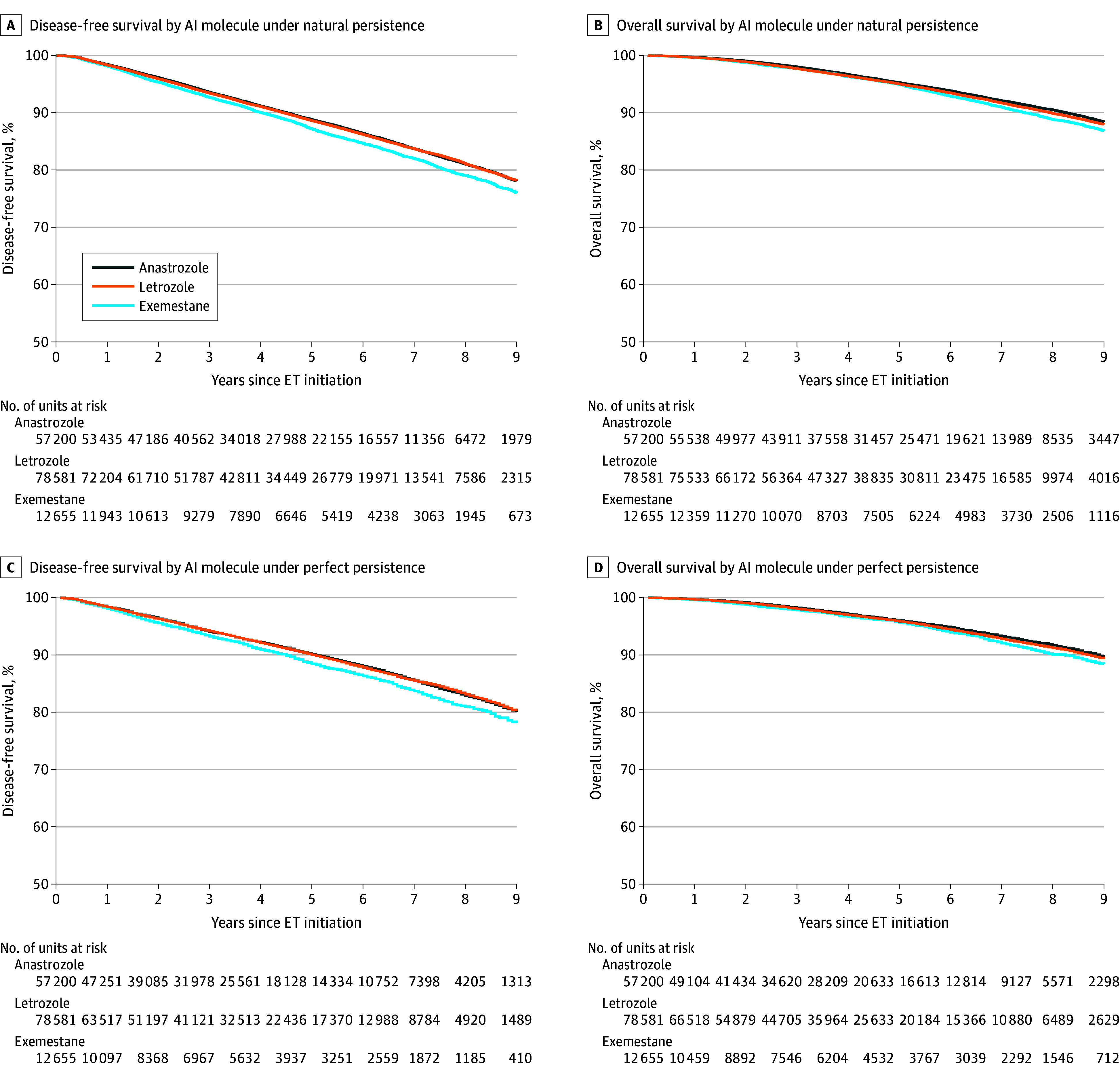
Disease-Free Survival and Overall Survival by Aromatase Inhibitor (AI) Molecule Under Natural and Perfect Persistence The number at risk reflects the unweighted population in panels A and B and the unweighted population after censoring for endocrine therapy discontinuation in panels C and D.

Under natural persistence, 5-year DFS proportions were 88.8% (95% CI, 88.5%-89.1%) for anastrozole, 88.6% (95% CI, 88.3%-88.9%) for letrozole, and 87.2% (95% CI, 86.5%-87.9%) for exemestane (Figure 2A, Table 2). After 8 years, the differences in DFS reached −2.0 (95% CI, −3.1 to −1.0) percentage points for exemestane vs letrozole (79.1% [95% CI, 78.1%-80.0%] vs 81.1% [95% CI, 80.7%-81.5%]) and −2.0 (95% CI, −3.0 to −0.9) percentage points for exemestane vs anastrozole (79.1% [95% CI, 78.1%-80.0%] vs 81.0% [95% CI, 80.6%-81.5%]) (eTable 2 in Supplement 1). OS proportions were similar for the 3 AI molecules up to 5 years (Figure 2B). After 8 years, OS was 88.8% (95% CI, 88.0%-89.6%) for exemestane compared with 90.5% (95% CI, 90.2%-90.8%) for anastrozole (difference, −1.7 [95% CI, −2.5 to −0.8] percentage points) and 89.9% (95% CI, 89.6%-90.2%) for letrozole (difference, −1.1 [95% CI, −1.9 to −0.2] percentage points).

**Table 2.** Disease-Free Survival and Overall Survival Proportions After 5 and 8 Years for 3 Aromatase Inhibitor Molecules Under Natural and Perfect Persistence

AI molecule	DFS (95% CI), %		OS (95% CI), %	
	5 y	8 y	5 y	8 y
Natural persistence				
Anastrozole	88.8 (88.5-89.1)	81.0 (80.6-81.5)	95.3 (95.1-95.5)	90.5 (90.2-90.8)
Letrozole	88.6 (88.3-88.9)	81.1 (80.7-81.5)	95.0 (94.9-95.2)	89.9 (89.6-90.2)
Exemestane	87.2 (86.5-87.9)	79.1 (78.1-80.0)	94.9 (94.5-95.4)	88.8 (88.0-89.6)
Perfect persistence				
Anastrozole	90.1 (89.7-90.4)	82.9 (82.2-83.3)	96.0 (95.8-96.2)	91.7 (91.4-92.1)
Letrozole	90.0 (89.6-90.2)	83.1 (82.5-83.5)	95.9 (95.7-96.0)	91.2 (90.9-91.6)
Exemestane	88.4 (87.5-89.1)	81.0 (79.7-82.0)	95.7 (95.3-96.1)	90.1 (89.2-91.0)

Abbreviations: AI, aromatase inhibitor; DFS, disease-free survival; OS, overall survival.

### DFS and OS Under Perfect Persistence

Under perfect persistence, 5-year DFS proportions were estimated to be 90.1% (95% CI, 89.7%-90.4%) for anastrozole, 90.0% (95% CI, 89.6%-90.2%) for letrozole, and 88.4% (95% CI, 87.5%-89.1%) for exemestane (Figure 2C). We estimated that exemestane also led to lower 8-year DFS compared with anastrozole and letrozole (Table 2). OS proportions were similar across molecules up to 5 years (Figure 2D) but were lower after 8 years for exemestane with 90.1% (95% CI, 89.2%-91.0%) compared with 91.7% (95% CI, 91.4%-92.1%) for anastrozole (difference, −1.6 [95% CI, −2.6 to −0.7] percentage points) and 91.2% (95% CI, 90.9%-91.6%) for letrozole (difference, −1.1 [95% CI, −2.1 to −0.2] percentage points).

### Comparisons With Previous RCT Results

**Figure 3.**
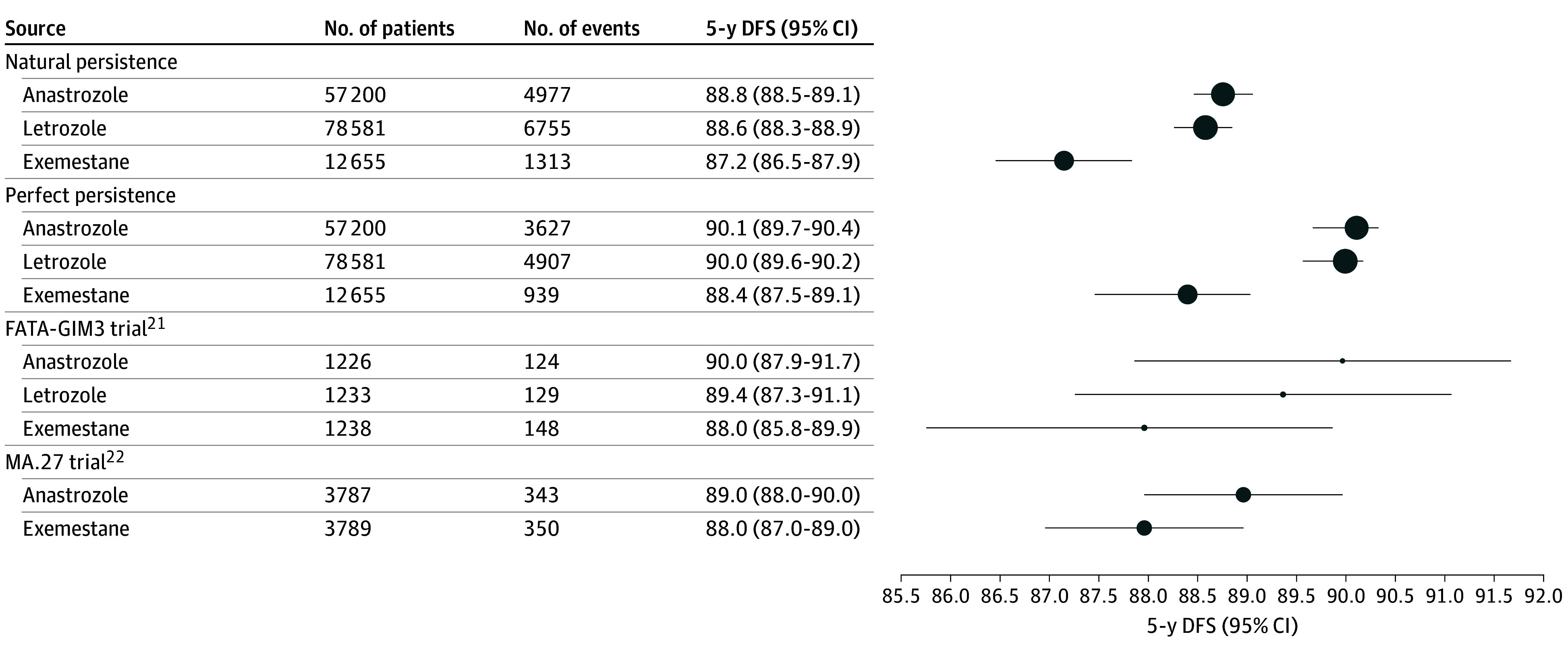
Comparison With Results From the FATA-GIM3 and MA.27 Randomized Clinical Trials The size of each point or circle is proportional to the logarithm of the number of patients. In the current study, the number of events refers to events occurring within the first 5 years of follow-up. For the MA.27 trial, the 5-year disease-free survival (DFS) was obtained from ClinicalTrials.gov, as only the 4-year DFS was reported in the primary publication. Error bars represent 95% CIs.

DFS proportion estimates observed at 5 years in the FATA-GIM3 and MA.27 RCTs were included between this study’s point estimates under natural persistence and perfect persistence for all 3 AI molecules (Figure 3). For example, the 5-year DFS for exemestane was 88.0% (95% CI, 85.8%-89.9%) in the FATA-GIM3 trial and 88% (95% CI, 87%-89%) in the MA.27 trial. This DFS was included in this study's estimates for exemestane under natural adherence (87.2%; 95% CI, 86.5%-87.9%) and perfect adherence (88.4%; 95% CI, 87.5%-89.1%). We present comparisons with FACE trial results for patients with node-positive disease in eFigure 1 in Supplement 1.

### Endocrine Therapy Discontinuation Risks and Switches

Adjusted 5-year endocrine therapy discontinuation risks were 35.1% (95% CI, 34.7%-35.6%) in the anastrozole group, 35.0% (95% CI, 34.6%-35.4%) in the letrozole group, and 39.3% (95% CI, 38.3%-40.3%) in the exemestane group (eFigure 2 in Supplement 1). In the anastrozole and letrozole groups, 25.4% and 27.7% of patients, respectively, switched to another endocrine therapy molecule at least once (eTable 3 and eFigure 3 in Supplement 1). For both drugs, the most common endocrine therapy molecule at first switch was exemestane, accounting for 52.4% and 58.7% of first switches, respectively. In the exemestane group, 28.9% of patients switched to another endocrine therapy molecule at least once: 27.3% switched to anastrozole, 41.9% to letrozole, and 30.8% to tamoxifen.

### Adverse Events

The risk of incident dyslipidemia at 5 years was 15.5% (95% CI, 14.7%-16.5%) in the exemestane group, 16.0% (95% CI, 15.6%-16.4%) in the anastrozole group, and 16.8% (95% CI, 16.3%-17.2%) in the letrozole group (eFigure 4 and eTable 4 in Supplement 1). The risk of diabetes and other adverse effects was slightly higher in the exemestane group than anastrozole group (diabetes: 9.0% [95% CI, 8.3%-9.6%] vs 8.0% [95% CI, 7.7%-8.3%]; other adverse effects: 9.8% [95% CI, 9.1%-10.5%] vs 8.8% [95% CI, 8.5%-9.1%]). The risk of bone fractures was similar across 3 AI molecules (both anastrozole and letrozole: 4.7% [95% CI, 4.5%-4.9%]; exemestane: 4.5% [95% CI, 4.0%-5.0%]).

### Sensitivity Analyses

The difference between exemestane and the 2 other AIs was increased in patients treated with chemotherapy. In these patients, OS at 8 years under perfect persistence was 84.8% (95% CI, 83.1%-86.6%) for exemestane compared with 88.4% (95% CI, 87.8%-89.2%) for anastrozole and 87.6% (95% CI, 87.0%-88.3%) for letrozole (eTable 5 in Supplement 1). Results from the other sensitivity analyses were similar to results in the main analysis.

## Discussion

We compared the clinical outcomes associated with adjuvant anastrozole, letrozole, and exemestane in a large cohort of women with postmenopausal HR-positive early-stage BC. We estimated that initiating exemestane led to slightly lower DFS and long-term OS (after 8 years) compared with both anastrozole and letrozole. Exemestane was also associated with higher discontinuation risks compared with anastrozole or letrozole. Overall, the treatment discontinuation proportions we observed were consistent with those reported in previous clinical studies, which have estimated 5-year discontinuation risks ranging from 31% to 73%.^28,36^ The large variability in previous estimates could be due to different data sources, definitions of discontinuation, or populations. Because imperfect persistence can increase the risk of BC recurrence and death,^37,38,39^ a natural hypothesis is that, with exemestane, lower persistence may contribute to worse outcomes. However, in our study, exemestane remained associated with lower DFS and OS even under perfect persistence, suggesting that persistence alone does not fully explain the observed differences.

The lower DFS and OS for exemestane may reflect underlying pharmacological differences among AIs.^12^ Prior studies suggested that letrozole is a more potent inhibitor of aromatization than exemestane and anastrozole.^14,40^ In a neoadjuvant setting, letrozole was shown to induce a more profound suppression of estrogens, with serum estrone and estradiol levels of 0.2 pmol/L and 0.4 pmol/L, respectively, compared with exemestane with levels of 1.8 pmol/L and 0.6 pmol/L, respectively.^15^ Similarly, in the adjuvant setting, letrozole produced greater suppression of plasma estrogens with serum estrone and estrone sulfate at 3 months than exemestane.^16^ Suboptimal estrogen suppression has been associated with higher risks of recurrence.^41^ Furthermore, exemestane has a corticosteroid backbone similar to testosterone, and its principal metabolite, 17β-hydroexemestane, behaves as a weak agonist of both estrogen receptor α (ERα) and androgen receptor (AR).^42^ In estrogen-deprived settings, these signals can stimulate proliferation of ERα- and AR-positive breast cells. Preclinical work in AI-resistant BC models has shown that AR activity collaborates with residual ERα signaling to sustain tumor growth despite aromatase blockade.^43^ Along with evidence of intratumoral androgen accumulation after exemestane therapy,^44^ these data support a model in which some ER-positive tumors transition from ER to AR dependence, thereby acquiring resistance to AIs. Taken together, existing evidence provides several possible biological explanations for our findings.

Regarding adverse events, our results were consistent with findings from previous studies,^16,17,45,46^ suggesting that steroidal exemestane could carry a slightly better lipid profile compared with anastrozole and letrozole. Higher androgenic signaling is associated with a more favorable lipid profile; thus, the mild androgenic activity of exemestane may account for its comparatively better lipid parameters.^47^ In the present study, the 5-year risk for bone fractures was consistent with the 4% reported in the FATA-GIM3 trial.^21^ Exemestane has been postulated to have a favorable bone profile due to its androgenic structure,^20^ but this hypothesis has not been confirmed in a companion analysis of the MA.27 trial.^48^ Similarly, the risk of fractures did not vary among the AI molecules in our analysis.

Our findings could be compared with those of the previous RCTs that conducted head-to-head comparisons of exemestane with another AI. The inclusion criteria in the FATA-GIM3^21^ and MA.27^22^ trials were broadly similar to this study’s criteria, except that patients older than 75 years were eligible in the 2 trials. Despite these extended eligibility criteria, the median age of participants in the trials was nearly identical to that in this study (64 years in the FATA-GIM3 trial, 64.3 years in the MA.27 trial, and 64 years in the present cohort). The 5-year DFS estimates reported in the FATA-GIM3 and MA.27 trials for each investigated AI were between our corresponding estimates under natural and perfect persistence, consistent with the expectation that persistence is higher in clinical trials than in clinical settings.^28^ Due to the large number of patients and events included in this cohort, the 95% CIs were much narrower than those reported in previous RCTs, allowing for the detection of small differences in outcomes that the FATA-GIM3 and MA.27 trials were underpowered to detect.

Using a target trial emulation framework,^34^ we designed this study to align closely with the previous RCTs, allowing for a comparison of estimated causal effects under explicit assumptions. Our cohort included 148 436 postmenopausal women with HR-positive early-stage BC, observed for more than a decade. The large sample size and extended follow-up allowed for the detection of differences in OS at 8 years that could not have been evidenced at 5 years due to the slow progression of BC.^4^

### Limitations

Despite adjusting for a wide range of covariates, unmeasured confounding cannot be entirely excluded. Cancer stage was unavailable. Nevertheless, the close agreement of our DFS estimates with previous RCT estimates supports the validity of our results. Moreover, because the choice of initial AI molecule in France is largely at the physician’s discretion, confounding by indication is likely to be limited. We assumed that patients consumed all dispensed medication, which could lead to underestimation of discontinuation risks and survival benefits under perfect persistence. However, this assumption would not bias our estimates under observed persistence. Because we emulated pragmatic strategies that allowed for treatment switches whenever needed, our results might not be transportable to populations with different distributions of treatment switches during follow-up. Finally, our results are specific to the upfront use of AIs and cannot be directly generalized to strategies involving tamoxifen followed by an AI given that such sequential strategies are rarely used in France.

## Conclusions

In this comparative effectiveness study using a target trial emulation framework, findings suggest that adjuvant endocrine therapy with exemestane may result in slightly lower DFS and OS compared with anastrozole and letrozole in patients with postmenopausal HR-positive early-stage breast cancer. While modest, the differences between these treatments are clinically relevant given the widespread use of AIs. In the absence of current guidelines for AI selection, our results favor anastrozole or letrozole as initial treatment. Furthermore, along with the recent adoption of AIs in combination with CDK4/6 inhibitors or ovarian suppression, the results highlight the need for future studies to inform AI selection across new treatment regimens and specific subpopulations.

## Notes

Supplement 1.
eMethods
**eTable 1.** List of CCAM, ICD-10, ATC, and LPP Codes Used to Identify ET Adverse Events and Side Effect Therapy **eTable 2.** Differences (in Percentage-Points) in Disease-Free Survival (DFS) and Overall Survival (OS) Proportions After 5 and 8 Years Along With Their 95% Confidence Intervals for Exemestane Versus Letrozole, Exemestane Versus Anastrozole, and Letrozole Versus Anastrozole Under Natural and Perfect Persistence **eTable 3.** Frequency and Distribution of First Treatment Switches per AI Molecule Under Natural Persistence **eTable 4.** 5-Year Incidence Risks of Adverse Event and 95% CI per AI Molecule Under Natural Persistence **eTable 5.** Results of the Sensitivity Analyses: DFS and OS at 5 and 8 Years Along With Their 95% Confidence Intervals Under Natural and Perfect Persistence **eFigure 1.** Number of Patients, Number of Events, and 5-Year Disease-Free Survival (DFS) With 95% Confidence Intervals for Anastrozole and Letrozole Under Observed and Perfect Persistence in Our Study for Patients With Node-Positive Disease (Proxy), Compared to Estimates From the FACE Randomized Controlled Trial **eFigure 2.** Risks of ET discontinuation for Anastrozole, Letrozole, and Exemestane Up to Five Years **eFigure 3.** Distribution of Treatment Changes and Discontinuations by Month for Patients Starting (A) Exemestane, (B) Letrozole, (C) Anastrozole **eFigure 4.** Cumulative Risks of Adverse Events Up to Five Years per AI Molecule Under Observed Persistence
eReferences

Supplement 2.
Data Sharing Statement
