# Supplementary material for: Outcomes of Anastrozole, Letrozole, and Exemestane in Patients With Postmenopausal Breast Cancer
Source: JAMA Netw Open. 2025 Dec 26;8(12):e2550842. doi: 10.1001/jamanetworkopen.2025.50842 (PMC12743288; doi:10.1001/jamanetworkopen.2025.50842)
Supplement: Supplement 1. — eMethods eTable 1. List of CCAM, ICD-10, ATC, and LPP Codes Used to Identify ET Adverse Events and Side Effect Therapy eTable 2. Differences (in Percentage-Points) in Disease-Free Survival (DFS) and Overall Survival (OS) Proportions After 5 and 8 Years Along With Their 95% Confidence Intervals for Exemestane Versus Letrozole, Exemestane Versus Anastrozole, and Letrozole Versus Anastrozole Under Natural and Perfect Persistence eTable 3. Frequency and Distribution of First Treatment Switches per AI Molecule Under Natural Persistence eTable 4. 5-Year Incidence Risks of Adverse Event and 95% CI per AI Molecule Under Natural Persistence eTable 5. Results of the Sensitivity Analyses: DFS and OS at 5 and 8 Years Along With Their 95% Confidence Intervals Under Natural and Perfect Persistence eFigure 1. Number of Patients, Number of Events, and 5-Year Disease-Free Survival (DFS) With 95% Confidence Intervals for Anastrozole and Letrozole Under Observed and Perfect Persistence in Our Study for Patients With Node-Positive Disease (Proxy), Compared to Estimates From the FACE Randomized Controlled Trial eFigure 2. Risks of ET discontinuation for Anastrozole, Letrozole, and Exemestane Up to Five Years eFigure 3. Distribution of Treatment Changes and Discontinuations by Month for Patients Starting (A) Exemestane, (B) Letrozole, (C) Anastrozole eFigure 4. Cumulative Risks of Adverse Events Up to Five Years per AI Molecule Under Observed Persistence eReferences [file jamanetwopen-e2550842-s001.pdf]

Supplemental Online Content

Dumas E, Hamy AS, Wanis KN, et al. Anastrozole, letrozole, and exemestane effectiveness in patients with postmenopausal breast cancer. *JAMA Netw Open*. 2025;8(12):e2550842. doi:10.1001/jamanetworkopen.2025.50842

eMethods

eTable 1. List of CCAM, ICD-10, ATC, and LPP Codes Used to Identify ET Adverse Events and Side Effect Therapy

eTable 2. Differences (in Percentage-Points) in Disease-Free Survival (DFS) and Overall Survival (OS) Proportions After 5 and 8 Years Along With Their 95% Confidence Intervals for Exemestane Versus Letrozole, Exemestane Versus Anastrozole, and Letrozole Versus Anastrozole Under Natural and Perfect Persistence

eTable 3. Frequency and Distribution of First Treatment Switches per AI Molecule Under Natural Persistence

eTable 4. 5-Year Incidence Risks of Adverse Event and 95% CI per AI Molecule Under Natural Persistence

eTable 5. Results of the Sensitivity Analyses: DFS and OS at 5 and 8 Years Along With Their 95% Confidence Intervals Under Natural and Perfect Persistence

eFigure 1. Number of Patients, Number of Events, and 5-Year Disease-Free Survival (DFS) With 95% Confidence Intervals for Anastrozole and Letrozole Under Observed and Perfect Persistence in Our Study for Patients With Node-Positive Disease (Proxy), Compared to Estimates From the FACE Randomized Controlled Trial

eFigure 2. Risks of ET discontinuation for Anastrozole, Letrozole, and Exemestane Up to Five Years

eFigure 3. Distribution of Treatment Changes and Discontinuations by Month for Patients Starting (A) Exemestane, (B) Letrozole, (C) Anastrozole

eFigure 4. Cumulative Risks of Adverse Events Up to Five Years per AI Molecule Under Observed Persistence

eReferences

This supplemental material has been provided by the authors to give readers additional information about their work.

© 2025 Dumas E et al. *JAMA Network Open*

eMethods

Data source and cohort inclusion/exclusion criteria

The FRESH database includes the SNDS data of all patients included in the FRESH cohort, which were identified with the Oncology Data Platform (ODP) available at the French National Cancer Institute (INCa). SNDS data has been described in detail elsewhere[1,2]. Briefly, it includes (i) demographic data (sex, date of birth, zip code of the town of residence, vital status, date of death if appropriate, health insurance regimen), (ii) hospital discharge reports (diagnoses, medical procedures, and expensive treatments), (iii) outpatient care (drugs dispensed, with the date of delivery, laboratory tests, and outpatient medical procedures) and (iv) long-term illness (LTI) records. At the time of analysis, demographic data, hospital discharge reports and outpatient care data were available for the year preceding patient inclusion, up to December 31, 2021. Data for the patients' histories of LTI were available from July 1, 2005, up to December 31, 2021.

The diagnosis codes were recorded in the SNDS, based on the International Classification of Diseases – 10th revision, ICD-10[3]. Procedures were recorded with the CCAM classification (*Classification Communes des Actes Médicaux*). Biological procedures were recorded with the NABM classification (*Nomenclature des Actes de Biologie Médicale*). Medications prescribed in outpatient care were recorded with CIP (*Code Identifiant de Présentation*) codes. In hospital, only costly innovative drugs included in a special reimbursement process called "*list en sus*" were recorded, in the form of UCD (*Unités Communes de Dispensation*) codes. Both the UCD and CIP codes were linked to the ATC classification (Anatomical Therapeutic and Chemical

© 2025 Dumas E et al. *JAMA Network Open*

classification) of the World Health Organization. Medical devices reimbursed by the French health insurance system (external prosthetics, orthotics, active implantable medical devices, invalid carriages, medical beds etc.) were recorded with LPP (*Liste des produits et prestations*) codes. In outpatient care, the type of medical service (teleconsultation, nursing care, dental care, etc.) was recorded with NGAP (*Nomemenclature Générale des Actes Professionnels*) codes.

We restricted our analysis to patients who underwent breast surgery. Eligibility was not based on receipt of chemotherapy, radiotherapy, or targeted therapy, which could have been administered in the neoadjuvant setting, the adjuvant setting, both, or neither. Patients who received neoadjuvant endocrine therapy were also included; however, all endocrine therapy-related variables were defined based on adjuvant treatment only.

We restricted our analysis to patients older than 50 and younger than 75 years at the time of BC diagnosis because: (1) our focus was on postmenopausal patients, (2) a systematic screening program is organized from age 50 to 75 years in France, (3) the frequency of non-cancer deaths in women ≥75 years would have required special considerations and interpretations for the treatment of death from other causes in our composite outcome, and (4) the risk/benefit of adjuvant endocrine therapy in elderly patients diagnosed with low risk early-stage breast cancer remains unknown.[4]

© 2025 Dumas E et al. *JAMA Network Open*

The “Régime General” covers approximately 70-80% of French citizens. The remaining citizens are still covered by the national health insurance system - as is every citizen in France- but under a different scheme. This means that their healthcare reimbursements are collected by a different agency. Citizens not covered by the general scheme include certain professions that have historically had separate schemes. These professions are diverse: railway workers, farmers, the military, the self-employed, the Assemblée Nationale (French Parliament) and the Senate workers. Access to healthcare is decided at the government level and is similar for all schemes. However, the death status may not be comprehensively collected for schemes other than the general scheme, particularly in the early years of the cohort. This is the reason why we limited our analysis to citizens enrolled in the general scheme.

Treatment of chronic diseases registered as long-term illness (LTI) is reimbursed at a 100% rate, without out-of-pocket expenses. This includes all three aromatase inhibitors as endocrine therapy for the treatment of breast cancer.

ET persistence

At a given time of follow-up, a patient was considered to have discontinued ET for more than 30 consecutive days (and consequently to be non-persistent) if the patient had not been dispensed ET in the previous 30 days and did not have an excess of pills from a previous dispensing that was expected to last beyond the start of the previous 30 days. We calculated the effective end date of pill supply from each dispensing by iteratively reporting the number of days supplied at each dispensing, starting from the effective end date of pill supply from the

© 2025 Dumas E et al. *JAMA Network Open*

previous dispensing. The number of days supplied at each dispensation is computed as the sum of pills included in the delivered boxes. We updated ET discontinuation status every 30 days after ET initiation, and up to 60*30 days after ET initiation.

We opted for a 30-day threshold in the definition of persistence because our objective is to compare counterfactual survival under perfect (ideal) persistence after initiation of one of the three AI drugs, thereby disentangling the intrinsic direct effects of the drugs from the indirect effects caused by the risk of discontinuing treatment. As patients cannot receive fewer than 30 pills in a single delivery, 30 days was the shortest possible threshold within our data.

Outcomes

We defined disease-free survival (DFS), as the absence of BC recurrence (including loco-regional recurrence, contralateral recurrence, or distant recurrence) and death. The occurrence of any of loco-regional recurrence, distant recurrence, or contralateral recurrence, was identified based on (i) the resumption of radiotherapy, chemotherapy, or targeted therapy after a gap of at least six months after the end of the initial treatments, (ii) a breast surgery procedure with axillary procedure performed at least 6 months after the end of the initial treatments, (iii) the intake of an anti-cancer molecule approved only in the metastatic setting starting at least six months after initial breast surgery, or (iv) the presence of a diagnosis code of metastasis in hospitalization stays starting at least six months after initial breast surgery. We defined the date of BC recurrence as the first day of any of the above events. The list of procedure, diagnosis, and medication codes is available elsewhere.[5] We did not include breast cancer-specific

© 2025 Dumas E et al. *JAMA Network Open*

survival in the evaluated endpoints because the FRESH database only had cause of death information for deaths occurring between 2013 and 2016.

No patient was lost-to-follow-up. All patients were administratively censored on 12/31/2021 for DFS and on 03/31/2022 for OS. Administrative censoring was considered non-informative.

The occurrence of an adverse event was detected on the basis of diagnosis (ICD10), procedure (CCAM), medical devices (LPP), and medication (ATC) codes. The list of codes can be found in eTable 1.

Covariates

Baseline covariates

Type of breast surgery: Breast surgery for BC was tagged with CCAM procedure codes at the hospital and were classified into two categories: (1) lumpectomy, and (2) mastectomy. The list of CCAM procedure codes can be found elsewhere[6]. The *index surgery* for BC was defined as the date on which the first breast surgical operation for BC took place, in the year of the first tag with a diagnosis code for BC. This date was used as a reference for the definition of the date of BC diagnosis, and for the other BC treatments, as explained below.

Age: We calculated age at BC diagnosis as the interval from birth to the BC diagnosis date rounded to the nearest year. The date of birth was directly available in the SNDS data. The date of BC diagnosis was taken as the date of either the earliest breast core biopsy in the year before

© 2025 Dumas E et al. *JAMA Network Open*

the index surgery, or the earliest fine-needle aspiration cytology, or the earliest breast imaging procedure or the date of the first BC treatment. Breast core biopsy, fine-needle aspiration cytology, and breast imaging procedure were tagged with CCAM procedure codes. The list of CCAM procedure codes can be found elsewhere[6]. We treated age as a continuous variable.

***HER2* status**: *Anti-HER2 (human epidermal growth factor receptor 2) targeted therapy sessions* were identified by ATC codes for trastuzumab and/or pertuzumab. Patients were considered to have received targeted therapy if they had at least one anti-*HER2* targeted therapy session between 250 days before and up to 180 days after BC index surgery, in accordance with clinical practices. Tumors of patients receiving anti-*HER2* targeted therapy were classified as *HER2*-positive; *HER2*-negative otherwise. Although the definitions of this variable could be based on data collected after the onset of ET, the type of treatment administered to the patient (anti-*HER2* therapy, radiotherapy and/or endocrine therapy) and the topological order of these treatments are decided immediately after BC surgery, depending on the disease biology and patient characteristics. Thus, we hypothesized that the initiation of anti-*HER2* targeted therapy, although possibly measured post-baseline, should not be influenced by AI intake. These arguments also hold for chemotherapy and radiotherapy status.

Nodal status: Lymph node involvement was tagged by the presence of at least one ICD-10 diagnosis code for node disease (C773) between 250 days before initial breast surgery and up to initial breast surgery. Nodal status was classified in a binary manner: “Node-positive” in presence of lymph node involvement; “Node-negative” otherwise.

Mammographic screening in the year preceding BC diagnosis: screening mammograms were identified based on the presence of the procedure code QEQK004 in either hospital and

outpatient care records in the year preceding BC diagnosis. The variable was binned into the following categories: 'yes' if the patient underwent a screening mammography in the year preceding BC diagnosis, or 'no' otherwise.

Type of medical structure: The type of medical structure was defined as the type of medical structure for the breast surgery. It was categorized into: (1) Comprehensive cancer centers ("Centre de lutte contre le cancer", CLCC), (2) Public hospitals, (3) Profit private hospitals, (4) Non-profit private hospitals.

Radiotherapy: Radiotherapy sessions were identified by ICD-10 diagnosis codes or CCAM procedure codes; and the use of radiotherapy was classified in a binary manner. A patient was considered to have been treated with radiotherapy if she had at least one radiotherapy session between 250 days before and up to 180 days after BC index surgery, in accordance with clinical practices. The list of ICD-10 diagnosis codes and CCAM procedure codes can be found elsewhere[6].

Chemotherapy: *Chemotherapy (CT) sessions* were identified by ICD-10 diagnosis codes, CCAM procedure codes and ATC molecule codes for hospital and outpatient care. The list of ICD-10 diagnosis codes, CCAM procedure codes and ATC molecule codes can be found elsewhere[6]. A patient was considered to have been treated with CT if she had at least one CT session between 250 days before and up to 180 days after BC index surgery, in accordance with clinical practices. The use of chemotherapy was classified in a binary manner.

Chemotherapy setting: Chemotherapy setting was tagged as: (1) adjuvant only if all chemotherapy sessions occurred after the BC index surgery date; (2) neoadjuvant +/- adjuvant otherwise.

© 2025 Dumas E et al. *JAMA Network Open*

Deprivation index: The area of residence was defined as the zip code of the town of residence of the patient at the time of first BC surgery. We used the 'FDep15' geographic socioeconomic index as a measure of social deprivation, as described elsewhere.[7] This index was defined for mainland France exclusively. The patient's deprivation index was set as the 'FDep15' index for the patient's area of residence in mainland France. It was set as 'missing' for overseas departments. The deprivation index was classified into six categories: (1) "Overseas departments"; and the five quintiles of the distribution for patients living in mainland France: (2) "1st quintile (least deprived)", (3) "2nd quintile", (4) "3rd quintile", (5) "4th quintile" and (6) 5th quintile (most deprived).

Type of comorbid conditions: At baseline (ET onset), the presence of a comorbid condition was detected on the basis of diagnosis (ICD10), procedure (CCAM), biology (NABM), medical devices (LPP), and medication (ATC) codes. The list of codes can be found in elsewhere[5]. A given comorbid condition was suspected at baseline for a patient if: (1) there was at least one biology, procedure, or medication code associated with the given comorbid condition in the previous year, (2) there was at least one LTI related to an ICD10 code associated with the given comorbid condition in the previous year, (3) there was at least one hospital discharge report containing an ICD10 diagnosis code associated with the comorbid condition concerned in the previous year, or (4) the comorbid condition considered was "Frailty (proxy)" and the patient had at least 150 days of home nursing care (NGAP code "AIS") in the previous year. In total, we identified 53 diseases as comorbid conditions, further grouped into 4 categories: (1) cardiovascular, (2) endocrine and metabolism, (3) psychiatric, and (4) other.

© 2025 Dumas E et al. *JAMA Network Open*

Number of comorbid conditions: The number of comorbid conditions was defined at the disease level (of the 53 comorbid conditions) and categorized into four categories: 0, 1, 2-4, and 5+.

Time-varying covariates

Adverse events: the occurrence of an adverse event at a given month was identified as described above (section Outcomes).

Anti-pain or anti-inflammatory drugs: At a given month of follow-up, the delivery of anti-pain or anti-inflammatory drugs was identified based on dispensations of any analgesics (ATC code: N02) or anti-inflammatory and antirheumatic product (ATC code: M01) during that month.

Vitamin D/calcium supplementation: At a given month of follow-up, the delivery of vitamin D/calcium supplementation was identified based on dispensations of any vitamin A and D in combination (ATC code: A11CB), vitamin D and analogues (ATC code: A11CC), or calcium (ATC code: A12A) during that month.

Causal assumptions

The identification of the counterfactual survival under arms 1-3 requires the causal assumptions of (i) consistency, (ii) positivity, and (iii) conditional exchangeability at baseline for the choice of the molecule. The identification of the counterfactual survival under arms 4-6 requires the causal assumption of (i) consistency, (ii) positivity, (iii) conditional

© 2025 Dumas E et al. *JAMA Network Open*

exchangeability at baseline for the choice of the molecule, and (iv) sequential exchangeability for treatment persistence.

Statistical analysis

The study population was described in terms of frequencies for qualitative variables, or medians and interquartile ranges (IQR) for quantitative variables. Median follow-up and its interquartile range (IQR) were assessed by reverse Kaplan-Meier methods. All confidence intervals were two-sided. After excluding patients with missing birthdates or areas of residence (less than 1% of the cohort, see inclusion/exclusion criteria and Figure 1), no missing data remained in the FRESH dataset.

Under strategies of arms 1,2,3 (natural persistence), patients could be censored at administrative censoring or if they experience the event only. Under strategies of arms 4,5,6 (perfect persistence), patients could further be censored due to non-persistence at the end of each 30-day period, provided that the patient had discontinued ET for more than 30 days at the end of the 30-day period.

In the analyses of arms 1,2,3 (natural persistence), IPT weights were estimated based on a multinomial log-linear model. First, we fitted a multinomial log-linear model to estimate the probability of initiating anastrozole, letrozole, or exemestane based on the observed baseline covariates. The model did not include interaction terms. The IPT weights are then computed as

© 2025 Dumas E et al. *JAMA Network Open*

the product of a stabilization term and the inverse of the probability of initiating the AI molecule the patient actually initialized. The stabilization term was computed as the frequency of patients initiating each molecule. The final weights were then truncated at the 99th upper percentile of the distribution.

In the analyses of arms 4,5,6 (perfect persistence), inverse probability of censoring (IPC) weights were estimated based on a pooled logistic regression model. First, we fitted a pooled logistic regression model to estimate the probability of being covered by ET treatment at a 30-day period based on the observed baseline and time-varying covariates. One model was fitted for each arm separately (anastrozole, letrozole, and exemestane). The models did not include interaction terms. The time-specific contributions to the weights at time t were computed as the inverse of the probability of being covered at time t as computed by the fitted weight model. IPC weights are then computed as the cumulative product of the time-specific contributions to the weights. We estimated IPT weights based on the same methodology than for arm 1,2, and 3. Finally, IPT/IPC final weights are computed as the product of the IPT weights and the IPC weights. Weights were updated from the start of ET until 60 months after the start of ET. The final weights were then truncated at the 99th percentile of the monthly distribution.

ISPOR Good Research Practices for Comparative Effectiveness Research

This study followed the ISPOR Good Research Practices for Comparative Effectiveness Research reporting guideline for nonrandomized studies.[8]

© 2025 Dumas E et al. *JAMA Network Open*

Study Design: We specified precisely the research question, target population, interventions, comparators, and outcomes. They were defined as closely as possible to those of the previously conducted randomized clinical trial FATA-GIM3.

Background: In the introduction we provide biological rationale and explain why the results from previous randomized clinical trials can be complemented and extended using large real-world dataset.

Data Source: We used the French Early Breast Cancer Cohort (FRESH), a nationwide database derived from comprehensive health insurance and hospital records. Data quality, completeness, and cohort were described in detail in the Methods section.

Study Population and Exposure Definition: The study population was clearly defined in the inclusion/exclusion criteria section and in the study flowchart. The hypothetical interventions considered were clearly defined. Observed treatment was identified from prescription dispensing records.

Outcome Definition: Primary outcomes were disease-free survival (DFS) and overall survival (OS). We clearly defined how the endpoints were measured on the data.

Confounding and analytical plan: We applied causal inference methods to adjust for measured confounding both at baseline and throughout follow-up (time-dependent). Sensitivity analyses assessed the robustness of results to assumptions about residual unmeasured confounding. We also showed that our results are consistent with those of the previous randomized clinical trials.

© 2025 Dumas E et al. *JAMA Network Open*

Transparency and Reproducibility: All analytic decisions, including inclusion/exclusion criteria, variable definitions, and modeling approaches, were prespecified. We provided sufficient detail, notably list of medical codes, to enable replication of the analysis using the same data source.

Interpretation and Limitations: Results were interpreted in light of potential residual confounding, data source limitations, and generalizability to other populations. We discussed the relevance of the findings to clinical decision-making.

Sensitivity analysis

Restriction to patients treated with chemotherapy

First, we restricted the population to patients treated with chemotherapy (neoadjuvant, adjuvant or both) and estimated DFS and OS under the six emulated arms as done in the main analysis. Use of chemotherapy served as a proxy of severe disease at BC diagnosis. Thus, if there really is a differential intrinsic effect of the three AIs, we would expect the effect to be of larger magnitude (on an absolute scale) for patients treated with chemotherapy than on the general population.

Restriction to patients treated in private hospitals

Second, we restricted the population to patients who underwent breast surgery in profit private hospitals, and estimated DFS and OS under the six emulated arms as done in the main analysis. The type of medical structure where the surgery was performed was associated with the AI molecule initiated at baseline, with patients initiating exemestane more frequently treated in

© 2025 Dumas E et al. *JAMA Network Open*

profit private hospitals (52.0%) compared with those who initiated anastrozole (41.8%) or letrozole (38.3%, Table 1). Since patients treated in private hospitals may have different socioeconomic profiles to those treated in other types of hospitals, and since we do not expect the effect of choosing an AI at baseline on disease-free survival to differ according to hospital type (*i.e,* no expected treatment effect heterogeneity), restricting the population to patients treated in private hospitals enables us to detect potential unmeasured socioeconomic confounding factors.

Restriction to patients not treated with neoadjuvant endocrine therapy

Third, we excluded patients treated with neoadjuvant endocrine therapy (n=1,653) from the analyses, and estimated DFS and OS under the six emulated arms as done in the main analysis. Patients starting endocrine therapy in the neoadjuvant setting constitute a particular subpopulation in our cohort because: (i) they may have specific tumor and characteristic profiles (*e.g.* older patients with smaller, less aggressive tumors); (ii) they may have already experienced side effects of endocrine therapy and discontinued treatment/changed AI drug before cancer surgery; (iii) they may interrupt endocrine therapy earlier than the rest of the population because they will reach the five-year treatment milestone sooner. Although they represented only a small proportion of the cohort, we conducted a sensitivity analysis excluding them to assess the reliance of our main results on this population.

© 2025 Dumas E et al. *JAMA Network Open*

Emulation of strategies of treatment enabling switches to tamoxifen but not to another AI

Fourth, we estimated DFS and OS under strategies of treatment where patients would be allowed to switch to tamoxifen but not to another AI during the five first years of treatment. Since we chose to emulate pragmatic interventions allowing patients to switch to another AI molecule whenever needed during treatment, our results are sensitive to the distribution and timing of treatment switches and reflect both the indirect effect through treatment switches and direct intrinsic effect of the drugs on disease-free survival. To alleviate this, we ran a sensitivity analysis where we emulated interventions where patients were allowed to switch to tamoxifen only, and not to another AI drug.

In the emulation of these alternative strategies for arm 1-3 (observed persistence), we censored patients at the first switch to another AI, if any. We then re-weighted the population. Weights were computed as the product of IPT weights accounting for the choice of the molecules at baseline (computed as previously), and IPC weights accounting for censoring due to switches to another AI. IPC weights were computed as described above for the IPC weights accounting for censoring due to treatment discontinuation.

In the emulation of these alternative strategies for arm 4-6 (perfect persistence), we censored patients at first treatment discontinuation and at first switches to another AI, whichever occurs first. We then re-weighted the population. Weights were computed as the product of IPT weights accounting for the choice of the molecules at baseline, IPC weights accounting for

© 2025 Dumas E et al. *JAMA Network Open*

censoring due to treatment discontinuation, and IPC weights accounting for censoring due to switches to another AI.

No weight stabilization and no weight truncation

Fifth, we did not stabilize and did not truncate the weights. Heuristically, weight stabilization and truncation might decrease the variance of the estimators while increasing the bias. Running the analyses without weight stabilization and truncation allows us to test the robustness of our results.

Software

Analyses were performed with R software. Pooled logistic regression models were fitted with the glm function. Multinomial log-linear model was fitted with the multinom function from the nnet library. Kaplan-Meier curves were estimated with the package survival.

Distribution of the weights

Observed persistence

After truncation, the estimated weights ranged from 0.70 to 1.32 (median 1.00, IQR 0.94-1.06) for anastrozole, from 0.86 to 1.28 (median 0.99, IQR 0.95-1.04) for letrozole, and from 0.53 to 1.32 (median 0.93, IQR 0.8-1.16) for exemestane.

© 2025 Dumas E et al. *JAMA Network Open*

Perfect persistence

After truncation, the estimated weights ranged from 0.70 to 2.43 (median 1.17, IQR 1.06-1.31) for anastrozole, from 0.87 to 2.43 (median 1.18, IQR 1.31-2.43) for letrozole, and from 0.53 to 2.43 (median 1.18, IQR 0.98-1.44) for exemestane.

© 2025 Dumas E et al. *JAMA Network Open*

***eTable 1*: List of CCAM, ICD-10, ATC, and LPP codes used to identify ET adverse events and side effect therapy.**

Category	Adverse event or side effects	Code type	Codes
Dyslipidemia	Dyslipidemia	ATC	C10AA01, C10AA02, C10AA03, C10AA04, C10AA05, C10AA06, C10AA07, C10AA08, C10AB01, C10AB02, C10AB03, C10AB04, C10AB05, C10AB06, C10AB07, C10AB08, C10AB09, C10AB10, C10AB11, C10AC01, C10AC02, C10AC03, C10AC04, C10AD01, C10AD02, C10AD03, C10AD04, C10AD05, C10AD06, C10AD52, C10AX01, C10AX02, C10AX03, C10AX05, C10AX06, C10AX07, C10AX08, C10AX09, C10AX10, C10AX11, C10AX12, C10AX13, C10AX14, C10BA01, C10BA02, C10BA03, C10BA04, C10BA05, C10BA06, C10BX01, C10BX02, C10BX03, C10BX04, C10BX05, C10BX06, C10BX07, C10BX08, C10BX09, C10BX10, C10BX11, C10BX12, C10BX13, C10BX14, C10BX15
		ICD10	E78, E780, E781, E782, E783, E784, E785, E786, E788, E789
Diabetes	Diabetes	ATC	A10AB01, A10AB02, A10AB03, A10AB04, A10AB05, A10AB06, A10AB30, A10AC01, A10AC02, A10AC03, A10AC04, A10AC30, A10AD01, A10AD02, A10AD03, A10AD04, A10AD05, A10AD06, A10AD30, A10AE01, A10AE02, A10AE03, A10AE04, A10AE05, A10AE06, A10AE30, A10AE54, A10AE56, A10AF01, A10BA01, A10BA02, A10BA03, A10BB01, A10BB02, A10BB03, A10BB04, A10BB05, A10BB06, A10BB07, A10BB08, A10BB09, A10BB10, A10BB11, A10BB12, A10BB31, A10BC01, A10BD01, A10BD02, A10BD03, A10BD04, A10BD05, A10BD06, A10BD07, A10BD08, A10BD09, A10BD10, A10BD11, A10BD12, A10BD13, A10BD14, A10BD15, A10BD16, A10BD17, A10BD18, A10BD19, A10BD20, A10BD21, A10BD22, A10BF01, A10BF02, A10BF03, A10BG01, A10BG02, A10BG03, A10BH01, A10BH02, A10BH03, A10BH04, A10BH05, A10BH06, A10BH07, A10BH51, A10BJ01, A10BJ02, A10BJ03, A10BJ04, A10BJ05, A10BJ06, A10BK01, A10BK02, A10BK03, A10BX01, A10BX02, A10BX03, A10BX04, A10BX05, A10BX06, A10BX07, A10BX08, A10BX14, A10XA01
		ICD10	E10, E100, E101, E102, E103, E104, E105, E106, E107, E108, E109, E11, E110, E111, E112, E113, E114, E115, E116, E117, E118, E119, E12, E120, E121, E122, E123, E124, E125, E126, E127, E128, E129, E13, E130, E131, E132, E133, E134, E135, E136, E137, E138, E139, E14, E140, E141, E142, E143, E144, E145, E146, E147, E148, E149, G590, G632, G730, G990, H280, H360, I792, L97, M142, M146, N083
Cardiovascular	Cardiac arrythmias	ICD10	I44, I45, I47, I48, I49
		ATC	C01B, C07

© 2025 Dumas E et al. *JAMA Network Open*

	Ischeamia or infarction	ICD10	I20, I21, I22, I23, I24, I25, I251, I254, I255, I256, I258, I259
		CCAM	DAFA002, DAFA008, DDAA002, DDAF001, DDAF002, DDAF003, DDAF004, DDAF005, DDAF006, DDAF007, DDAF008, DDAF009, DDAF010, DDFF001, DDFF002, DDMA002, DDMA003, DDMA004, DDMA005, DDMA006, DDMA007, DDMA008, DDMA009, DDMA010, DDMA011, DDMA012, DDMA013, DDMA014, DDMA015, DDMA016, DDMA017, DDMA018, DDMA019, DDMA020, DDMA021, DDMA022, DDMA023, DDMA024, DDMA025, DDMA026, DDMA027, DDMA028, DDMA029, DDMA030, DDMA031, DDMA032, DDMA033, DDMA034, DDMA035, DDMA036, DDMA037, DDMA038, DDPF002, DDQF001, DDQH001, DDQH004, DDQH005, DDQH006, DDQH008, DDQH011, DDQH013, DDQH014, DDQH015, DDQM001
	Hypertension	ICD10	I10, I120, I129, I130, I131, I132, I139, I12, I13
		ATC	C02AB02, C02AC01, C02AC02, C02AC05, C02AC06, C02CA01, C02CA06, C02DC01, C02LA01, C03AA01, C03AA03, C03BA04, C03BA10, C03BA11, C03BX03, C03CA01, C03CA02, C03CA03, C03DA01, C03DB01, C03EA01, C03EA04, C08CA01, C08CA02, C08CA03, C08CA04, C08CA05, C08CA08, C08CA09, C08CA11, C08CA13, C08GA02, C09AA01, C09AA02, C09AA03, C09AA04, C09AA05, C09AA06, C09AA07, C09AA08, C09AA09, C09AA10, C09AA13, C09AA15, C09AA16, C09BA01, C09BA02, C09BA03, C09BA04, C09BA05, C09BA06, C09BA07, C09BA09, C09BA15, C09BB02, C09BB04, C09BB10, C09CA01, C09CA02, C09CA04, C09CA05, C09CA06, C09CA07, C09CA08, C09DA01, C09DA02, C09DA03, C09DA04, C09DA06, C09DA07, C09DA08, C09DB01, C09DB02, C09DB04, C09DB05, C09XA02, C09XA52, C10BX03, C03AA08, C03BA03, C03DB02
	CNS cerebrovascular ischaemia	ICD10	G45, G450, G451, G452, G453, G454, G458, G459, H340, H341, H342, I63, I630, I631, I632, I633, I634, I635, I636, I638, I639, I64, I69, I693, I694, I60, I61,I62
		CCAM	DGCA032, EAAF002, EAAF004, EAAF900, EAAF901, EAAF902, EAAF903, EAFA001, EANF002, EBAA002, EBAF001, EBAF002, EBAF003, EBAF004, EBAF005, EBAF006, EBAF007, EBAF008, EBAF009, EBAF010, EBAF011, EBAF013, EBAF014, EBCA001, EBCA002, EBCA004, EBCA005, EBCA008, EBCA010, EBCA011, EBCA013, EBCA014, EBCA015, EBCA017, EBEA002, EBEA003, EBEA004, EBEA005, EBFA002, EBFA003, EBFA005, EBFA006, EBFA008, EBFA009, EBFA010, EBFA012, EBFA014, EBFA015, EBFA016, EBFA017, EBFA018, EBFA019, EBFA020, EBFA021, EBKA001, EBKA002, EBKA003, EBKA004, EBNF001, EBNF002, ECAF003, ECAF004, ECFA001, ECLF004
	thrombolism or embolism	ICD10	I26, I80, I800, I801, I802, I803, I808, I809, I81, I82

© 2025 Dumas E et al. *JAMA Network Open*

Osteoporosis	Osteoporosis	ICD10	M80, M81, M82, M83
		ATC	M05B
Bone fracture	Bone fracture	ICD10	M80, S02, S12, S22, S32, S42, S52, S62, S72, S82, S92, M84, M907, M966, T02, T08, T10, T12, T142
		CCAM	LACA001, LACA002, LACA003, LACA004, LACA005, LACA006, LACA007, LACA008, LACA009, LACA010, LACA011, LACA013, LACA014, LACA015, LACA016, LACA017, LACA018, LACA019, LACA020, LACB001, LACB002, LACB003, LAEA001, LAEA003, LAEA005, LAEA007, LAEA008, LAEB001, LAEP001, LAEP002, LBCA001, LBCA002, LBCA003, LBCA004, LBCA005, LBCA006, LBCA007, LBCA008, LBCA009, LBCA01, LBCB001, LBCB002, LBED001, LBED002, LBED003, LBED004, LBED005, LBED006, LBEP002, LBEP009, LBLD002, LGCA001, LJCA001, MACA001, MACA002, MACA003, MACA004, MACB001, MACB002, MADP001, MAEP001, MBCA001, MBCA003, MBCA004, MBCA005, MBCA006, MBCA007, MBCA008, MBCA009, MBCA010, MBCA011, MBCA012, MBCB001, MBCB002, MBCB003, MBCB004, MBEB001, MBEP001, MBEP002, MBEP003, MCCA001, MCCA003, MCCA004, MCCA005, MCCA007, MCCA008, MCCA009, MCCA010, MCCA011, MCCB001, MCCB002, MCCB003, MCCB004, MCCB005, MCCB007, MCCB008, MCEP001, MCEP002, MDCA001, MDCA003, MDCA004, MDCA006, MDCA007, MDCA008, MDCA009, MDCA010, MDCA011, MDCA012, MDCA013, MDCA014, MDCB002, MDCB003, MDCB004, MDCB005, MDEP001, MDEP002, MDEP003, MEEA001, MEEA002, MEEP001, MEEP003, MFEA002, MFEA003, MFEB001, MFEP002, MGEA001, MGEP002, MHEA002, MHEA004, MHEP001, MHEP002, MHEP003, MHEP004, MZMP002, MZMP004, MZMP007, MZMP013, NACA001, NACA002, NACA003, NACA004, NACA005, NACB001, NAEP001, NAEP002, NBCA001, NBCA002, NBCA003, NBCA005, NBCA006, NBCA007, NBCA008, NBCA009, NBCA010, NBCA012, NBCA013, NBCA014, NBCA015, NBCB001, NBCB002, NBCB004, NBCB006, NBEB001, NBEP001, NBEP002, NCCA001, NCCA002, NCCA003, NCCA004, NCCA005, NCCA006, NCCA007, NCCA008, NCCA010, NCCA011, NCCA012, NCCA013, NCCA014, NCCA015, NCCA016, NCCA017, NCCA018, NCCA019, NCCB001, NCCB002, NCCB004, NCCB005, NCCB006, NCCB007, NCCC001, NCEP001, NCEP002, NDCA001, NDCA002, NDCA003, NDCA004, NDCA005, NDCA006, NDCB001, NDCB002, NDCB003, NDCB004, NDEP001, NEEA003, NEEP005, NFEP002, NGEP001, NZMP006, NZMP008, NZMP014
Depression or anxiety	Depression or anxiety	ICD10	F320, F321, F322, F323, F324, F325, F328, F329, F330, F331, F332, F333, F334, F338, F339, F341, F32, F33, F40, F41

© 2025 Dumas E et al. *JAMA Network Open*

		ATC	N03AA30, N05BA01, N05BA02, N05BA03, N05BA04, N05BA05, N05BA06, N05BA07, N05BA08, N05BA09, N05BA10, N05BA11, N05BA12, N05BA13, N05BA14, N05BA15, N05BA16, N05BA17, N05BA18, N05BA19, N05BA21, N05BA22, N05BA23, N05BA24, N05BA56, N05BB01, N05BB02, N05BB51, N05BC01, N05BC03, N05BC04, N05BC51, N05BD, N05BE01, N05BX01, N05BX02, N05BX03, N05BX04, N05BX05, N05CD01, N05CD02, N05CD03, N05CD04, N05CD05, N05CD06, N05CD07, N05CD09, N05CD10, N05CD11, N05CD12, N05CD13, N05CD14, N05CD15, N05CE, N05CF03, N05CF04, N05CH, N05CM01, N05CM02, N05CM03, N05CM04, N05CM06, N05CM07, N05CM08, N05CM09, N05CM10, N05CM11, N05CM12, N05CM13, N05CM15, N05CM16, N05CM18, N05CM19, N05CX, N05AA, N05CM
Gastrointestinal side effects	Gastrointestinal side ef-fects	ICD10	K25, K26, K27, K28, K290, K50, K51, K85, B252, K860, K861, M074, M075, K21, K29, K520
		ATC	A02, A03, A04, A05, A06, A09
Dermatologic	Dermatologic	ICD10	L29
		ATC	D04, D07
Other	Lymphatics (oedema)	ICD10	I890, Q820
		LPP	1302967, 1330277, 1351173, 1340152, 1349058, 1335323, 1390977, 1378410, 1367961, 1387202, 1324791, 1324213, 1381286, 1306310, 1370638, 1367412, 1338557, 1352020, 1353887, 1346574, 1314746, 1359714, 1312598, 1352741, 1328518, 1387030, 1326689, 1313238, 1314769, 1332320, 1314887, 1319956, 1352190
	Arthritis and arthroses	ICD10	M07, M070, M071, M072, M073, M074, M075, M076, M088, M089, M09, M15, M46, M47, M48, M49, M701, M702, M703, M706
	Vaginal estrogen therapy	ATC	G03JA05, G03JA06
	Endometrial cancer	ICD10	C54, C540, C541, C542, C543, C548, C549

© 2025 Dumas E et al. *JAMA Network Open*

Abbreviations: ICD 10: international statistical classification and related health problems – 10th revision; CCAM: “Classification Communes des Actes Médicaux”; ATC: anatomical therapeutic and chemical classification, LPP: “Liste des produits et prestations”; CNS: central nervous system.

© 2025 Dumas E et al. *JAMA Network Open*

Persis-tence	Molecules	DFS		OS	
		5 years	8 years	5 years	8 years
Natural	exemestane *versus* letrozole	-1.4 (-2.2 ; -0.7)	-2 (-3.1 ; -1)	-0.1 (-0.6 ; 0.4)	-1.1 (-1.9 ; -0.2)
	exemestane *versus* anastrozole	-1.6 (-2.3 ; -0.9)	-2 (-3 ; -0.9)	-0.3 (-0.8 ; 0.1)	-1.7 (-2.5 ; -0.8)
	letrozole *versus* anastrozole	-0.2 (-0.6 ; 0.2)	0.1 (-0.5 ; 0.7)	-0.2 (-0.5 ; 0)	-0.6 (-1.1 ; -0.2)
Perfect	exemestane *versus* letrozole	-1.6 (-2.5 ; -0.7)	-2.2 (-3.3 ; -0.9)	-0.2 (-0.6 ; 0.3)	-1.1 (-2.1 ; -0.2)
	exemestane *versus* anastrozole	-1.7 (-2.5 ; -0.9)	-1.9 (-3.1 ; -0.7)	-0.3 (-0.8 ; 0.2)	-1.6 (-2.6 ; -0.7)
	letrozole *versus* anastrozole	-0.1 (-0.6 ; 0.3)	0.2 (-0.5 ; 0.9)	-0.1 (-0.4 ; 0.1)	-0.5 (-1.1 ; 0)

eTable 2: Differences (in percentage-points) in disease-free survival (DFS) and overall survival (OS) proportions after 5 and 8 years along with their 95% confidence intervals for exemestane versus letrozole, exemestane versus anastrozole, and letrozole versus anastrozole under natural and perfect persistence.

Abreviations: DFS: disease-free survival; OS: overall survival.

© 2025 Dumas E et al. *JAMA Network Open*

Molecule at ET onset		anastrozole	letrozole	exemestane
**At least one molecule switch**	**No**	42,692 (74.6%)	56,820 (72.3%)	8,996 (71.1%)
	**Yes**	14,508 (25.4%)	21,761 (27.7%)	3,659 (28.9%)
First molecule after switch	*anastrozole*	-	*5,305 (24.4%)*	*999 (27.3%)*
	letrozole	*4,661 (32.1%)*	-	*1,533 (41.9%)*
	exemestane	*7,599 (52.4%)*	*12,768 (58.7%)*	-
	tamoxifen	*2,248 (15.5%)*	*3,688 (16.9%)*	*1,127 (30.8%)*

eTable 3: **Frequency and distribution of first treatment switches per AI molecule under natural persistence.**

Patients were considered to have switched treatment if, and when, they were delivered in pharmacy one of the other AIs or tamoxifen within five years of the first AI molecule initiation. This applied regardless of how long they had been on the initial molecule and how long they stayed on the switch molecule. The percentages of first molecule after switch are presented only for the subset of patients who switched treatment at least once. Abbreviations: ET: endocrine therapy.

© 2025 Dumas E et al. *JAMA Network Open*

	5-year incidence risk (%)		
**Adverse event**	**anastrozole**	**letrozole**	**exemestane**
Dyslipidemia	16 (15.6 - 16.4)	16.8 (16.3 - 17.2)	15.5 (14.7 - 16.5)
Diabetes	8 (7.7 - 8.3)	8.6 (8.3 - 8.8)	9 (8.3 - 9.6)
Cardiovascular	26.8 (26.2 - 27.4)	26.4 (25.9 - 26.9)	26.6 (25.5 - 28)
Osteoporosis	17.5 (17.2 - 17.9)	17.3 (17 - 17.6)	17.2 (16.4 - 18)
Bone fracture	4.7 (4.5 - 4.9)	4.7 (4.5 - 4.9)	4.5 (4 - 5)
Depression or anxiety	34.4 (33.8 - 35.1)	33.5 (32.9 - 34.1)	34.9 (33.5 - 36.4)
Gastrointestinal	76.5 (75.7 - 77.3)	75.6 (74.8 - 76.4)	77.7 (76 - 79.3)
Dermatologic	45 (44.4 - 45.5)	45 (44.5 - 45.5)	46.3 (45.1 - 47.6)
Other	8.8 (8.5 - 9.1)	9.3 (9 - 9.5)	9.8 (9.1 - 10.5)

eTable 4: **5-year incidence risks of adverse event and 95% CI per AI molecule under natural persistence.**

Analyses were restricted to patients without a history of the respective event in the year preceding AI initiation.

© 2025 Dumas E et al. *JAMA Network Open*

Sensitivity analysis	Persistence	Molecule	DFS		OS	
			5 years	8 years	5 years	8 years
(1) Population restriction to patients treated with chemotherapy	Natural	anastrozole	83.9 (83.3 - 84.4)	75.3 (74.6 - 76.1)	93 (92.6 - 93.4)	86.8 (86.3 - 87.5)
		letrozole	83.7 (83.2 - 84.2)	74.9 (74.2 - 75.7)	92.6 (92.2 - 92.9)	86.1 (85.5 - 86.7)
		exemestane	81.4 (80 - 82.8)	71.7 (70 - 73.5)	92 (91 - 92.9)	83.4 (82 - 84.8)
	Perfect	anastrozole	85.6 (84.8 - 86)	77.2 (76.1 - 78)	93.9 (93.5 - 94.3)	88.4 (87.8 - 89.2)
		letrozole	85.4 (84.7 - 85.8)	77 (76 - 77.8)	93.8 (93.5 - 94.2)	87.6 (87 - 88.3)
		exemestane	82.9 (81.1 - 84.2)	73.9 (71.5 - 75.9)	92.9 (92 - 94)	84.8 (83.1 - 86.6)
(2) Population restriction to patients treated in profit private hospitals	Natural	anastrozole	88.5 (88.1 - 89)	81.2 (80.5 - 81.9)	95.3 (95 - 95.6)	90.8 (90.3 - 91.3)
		letrozole	88.6 (88.2 - 89)	81.2 (80.6 - 81.8)	95.2 (94.9 - 95.5)	90.2 (89.8 - 90.7)
		exemestane	87.1 (86.2 - 88.1)	79.9 (78.6 - 81.2)	95.3 (94.7 - 95.9)	89.9 (88.9 - 90.8)
	Perfect	anastrozole	90 (89.4 - 90.3)	83.3 (82.3 - 84)	96 (95.7 - 96.4)	92 (91.5 - 92.6)
		letrozole	89.9 (89.3 - 90.2)	82.9 (82.1 - 83.5)	96 (95.7 - 96.3)	91.2 (90.7 - 91.8)
		exemestane	88 (86.8 - 89)	81.8 (80.3 - 83.3)	95.8 (95.3 - 96.5)	91.3 (90.3 - 92.4)
(3) Exclusion of patients treated with neoadjuvant endocrine therapy	Natural	anastrozole	88.9 (88.6 - 89.2)	81.2 (80.8 - 81.7)	95.3 (95.1 - 95.5)	90.6 (90.3 - 91)
		letrozole	88.8 (88.5 - 89.1)	81.4 (80.9 - 81.8)	95.1 (94.9 - 95.3)	90.1 (89.8 - 90.4)
		exemestane	87.4 (86.7 - 88.1)	79.4 (78.5 - 80.3)	95 (94.6 - 95.5)	89 (88.3 - 89.7)
	Perfect	anastrozole	90.2 (89.8 - 90.5)	83 (82.4 - 83.4)	96.1 (95.9 - 96.3)	91.8 (91.5 - 92.2)
		letrozole	90.2 (89.8 - 90.4)	83.4 (82.8 - 83.8)	96 (95.8 - 96.2)	91.4 (91 - 91.8)
		exemestane	88.7 (87.8 - 89.3)	81.2 (79.9 - 82.2)	95.8 (95.4 - 96.3)	90.2 (89.4 - 91)
(4) Treatment strategies authorizing switches to tamoxifen but not to another AI	Natural	anastrozole	89.8 (88.7 - 89.4)	82.5 (80.9 - 81.9)	96.2 (96.7 - 97)	91.4 (91.7 - 92.4)
		letrozole	89.9 (88.8 - 89.4)	82.9 (81.1 - 82)	95.8 (96.6 - 96.9)	90.6 (91.2 - 91.8)
		exemestane	88.2 (86.4 - 88)	80.5 (78.1 - 80.3)	95.6 (96.1 - 96.9)	89.5 (89.6 - 91.2)

© 2025 Dumas E et al. *JAMA Network Open*

	Perfect	anastrozole	90.5 (90.2 - 90.9)	83.3 (82.7 - 83.9)	97.3 (97.1 - 97.5)	92.9 (92.5 - 93.4)
		letrozole	91 (90.7 - 91.3)	84 (83.5 - 84.6)	97.4 (97.2 - 97.5)	92.7 (92.3 - 93.1)
		exemestane	89.2 (88.3 - 90)	81.9 (80.6 - 83.2)	97.2 (96.7 - 97.5)	91.6 (90.7 - 92.5)
(5) No weight stabilization and no weight truncation	Natural	anastrozole	88.8 (88.5 - 89.1)	81 (80.6 - 81.5)	95.3 (95.1 - 95.5)	90.5 (90.2 - 90.8)
		letrozole	88.6 (88.3 - 88.9)	81.1 (80.7 - 81.5)	95 (94.9 - 95.2)	89.9 (89.6 - 90.2)
		exemestane	87.1 (86.3 - 87.8)	79 (77.9 - 80)	95 (94.5 - 95.4)	88.8 (88 - 89.6)
	Perfect	anastrozole	90.1 (89.7 - 90.4)	82.9 (82.2 - 83.3)	96 (95.8 - 96.2)	91.7 (91.4 - 92.1)
		letrozole	90 (89.6 - 90.2)	83.1 (82.5 - 83.5)	95.8 (95.7 - 96)	91.2 (90.9 - 91.6)
		exemestane	88.3 (87.4 - 89)	80.9 (79.7 - 81.9)	95.7 (95.3 - 96.2)	90.1 (89.2 - 91)

eTable 5: **Results of the sensitivity analyses. DFS and OS at 5 and 8 years along with their 95% confidence intervals under natural and perfect persistence**.

Abbreviations: DFS: disease-free survival; OS: overall survival, AI: aromatase inhibitor.

© 2025 Dumas E et al. *JAMA Network Open*

Study	Persistence	Molecule	No. patients	No. events	5–year DFS (95% CI)
**Current study**	**Natural**	anastrozole	10,676	1,554	81 (80.2 – 81.8)
		letrozole	16,260	2,240	81.4 (80.7 – 82.2)
	**Perfect**	anastrozole	10,676	1,175	83 (81.8 – 83.6)
		letrozole	16,260	1,643	83.7 (82.7 – 84.3)
**FACE**		anastrozole	2,075	368	82.9 (81.2 – 84.5)
		letrozole	2,061	341	84.9 (83.2 – 86.4)

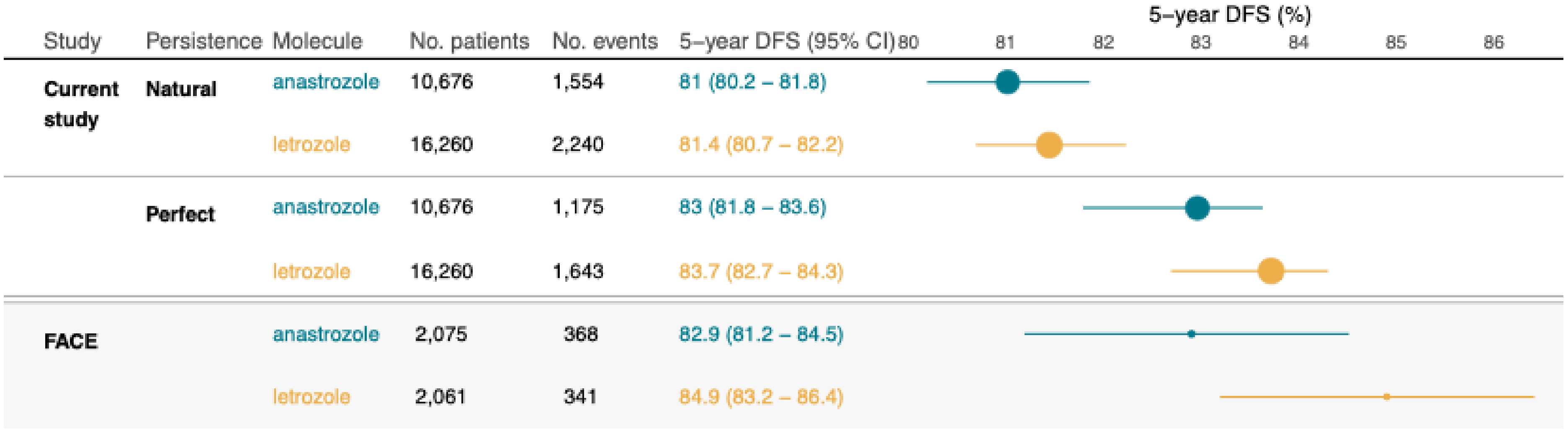

eFigure 1: **Number of patients, number of events, and 5-year disease-free survival (DFS) with 95% confidence intervals for anastrozole and letrozole under observed and perfect persistence in our study for patients with node-positive disease (proxy), compared to estimates from the FACE randomized controlled trial**.

The size of each point is proportional to the logarithm of the number of patients. *Abbreviations*: RCT: randomized controlled trial; DFS: disease-free survival

© 2025 Dumas E et al. *JAMA Network Open*

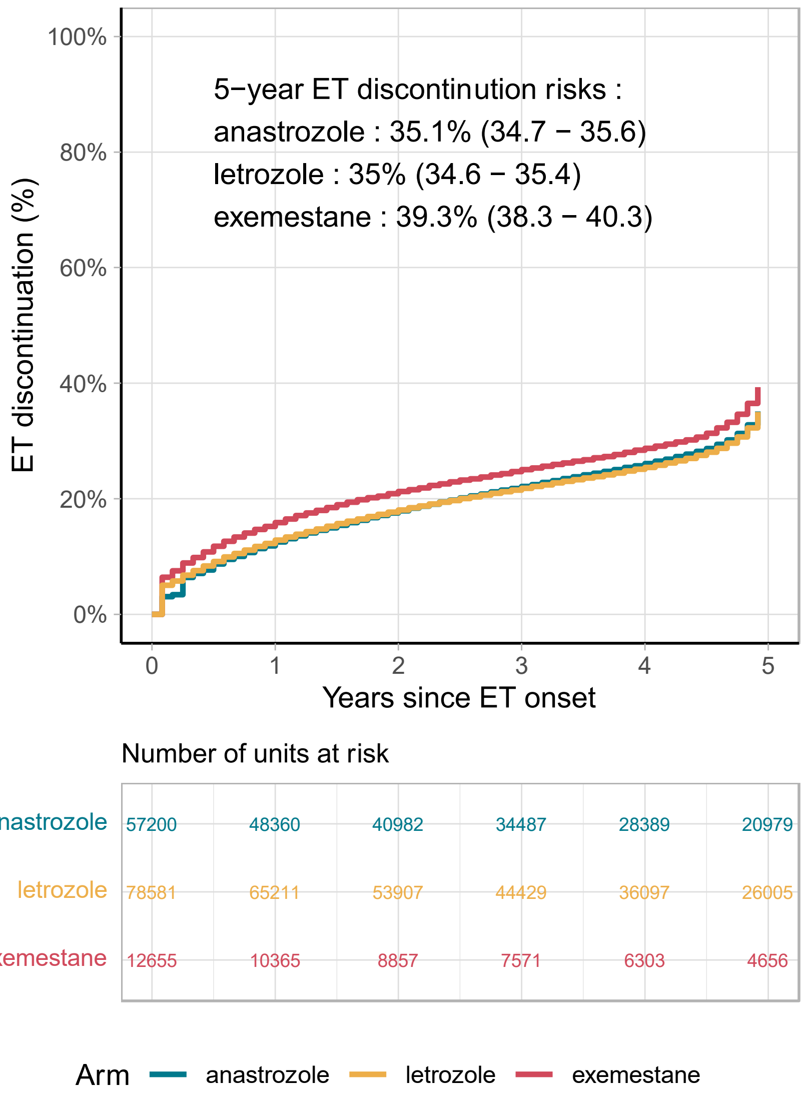

eFigure 2: **Risks of ET discontinuation for anastrozole, letrozole, and exemestane up to five years.**

The cumulative risk of ET discontinuation was estimated using Kaplan-Meier cumulative event curves fitted on the weighted populations of arm 1-3 (i.e. after adjustment for the choice of the AI molecule at baseline). The number at risk in the risk tables reflects the unweighted population. Abbreviations: ET: endocrine therapy.

© 2025 Dumas E et al. *JAMA Network Open*

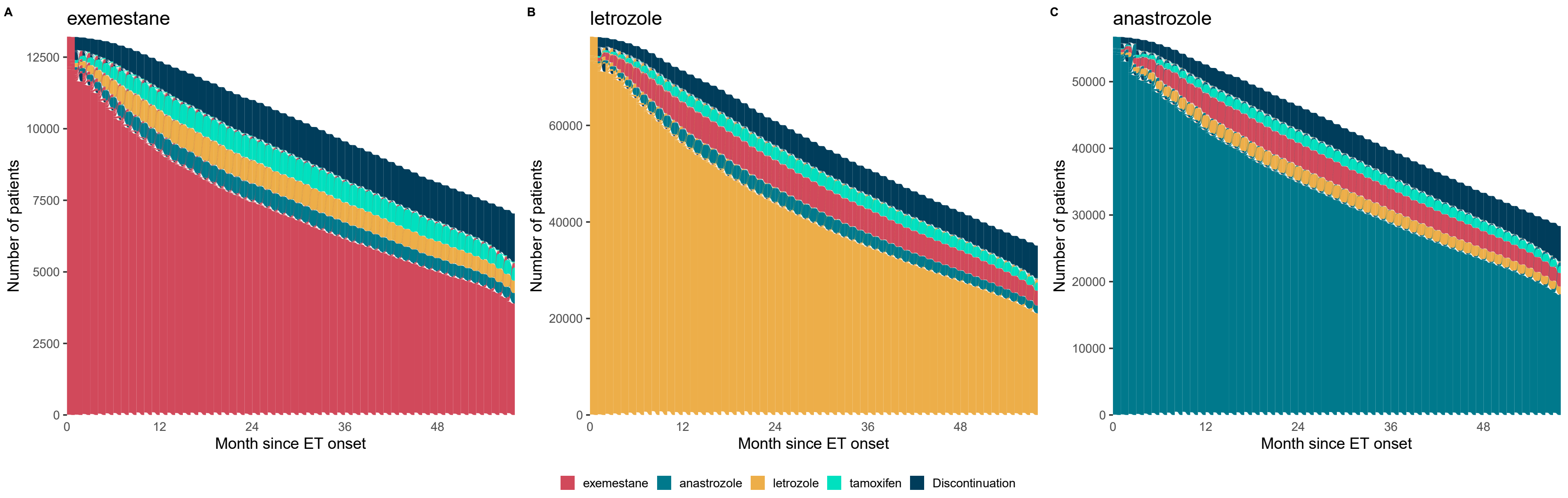

eFigure 3: **Distribution of treatment changes and discontinuations by month for patients starting (A) exemestane, (B) letrozole, (C) anastrozole.**

If the patient was covered by multiple molecules in a given month, we considered the patient to be covered by exemestane, then letrozole, then anastrozole, then tamoxifen. The number of patients decreases over time due to patients being administratively censored, recurring, or dying (white space on top).

© 2025 Dumas E et al. *JAMA Network Open*

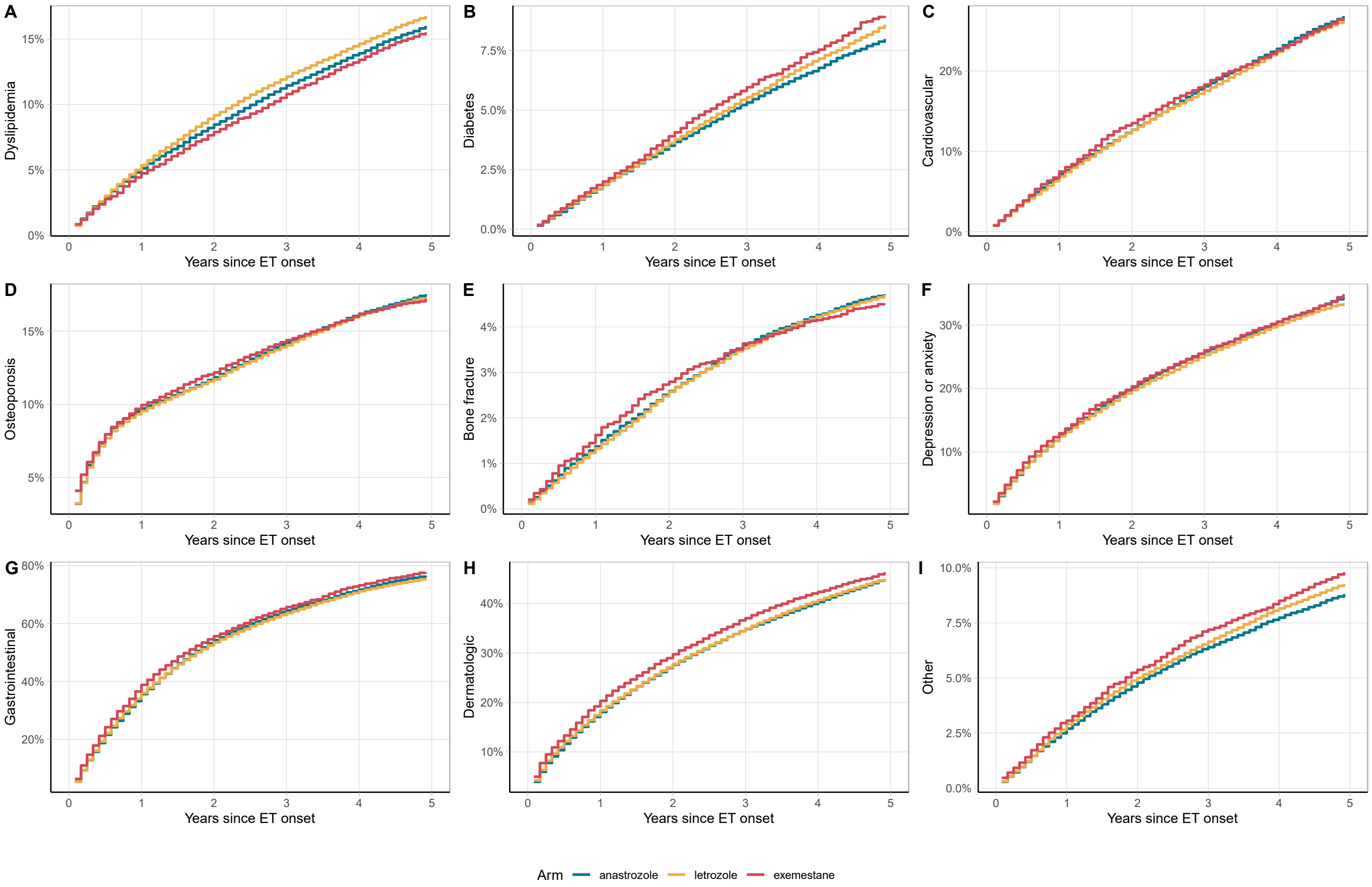

© 2025 Dumas E et al. *JAMA Network Open*

eFigure 4: **Cumulative risks of adverse events up to five years per AI molecule under observed persistence**.

Analyses were restricted to patients without a history of the respective event in the year preceding AI initiation. Abbreviations: ET: endocrine therapy.

© 2025 Dumas E et al. *JAMA Network Open*

eReferences

1. Tuppin P, Rudant J, Constantinou P, et al. Value of a national administrative database to guide public decisions: From the système national d'information interrégimes de l'Assurance Maladie (SNIIRAM) to the système national des données de santé (SNDS) in France. *Rev Epidemiol Sante Publique*. 2017;65 Suppl 4:S149-S167. doi:10.1016/j.respe.2017.05.004

2. Bousquet PJ, Lefeuvre D, Tuppin P, et al. Cancer care and public health policy evaluations in France: Usefulness of the national cancer cohort. *PLoS One*. 2018;13(10):e0206448. doi:10.1371/journal.pone.0206448

3. World Health Organization. International Classification of Diseases (ICD). Accessed January 20, 2022. https://www.who.int/standards/classifications/classification-of-diseases

4. Meattini I, Santis MCD, Visani L, et al. Single-modality endocrine therapy versus radiotherapy after breast-conserving surgery in women aged 70 years and older with luminal A-like early breast cancer (EUROPA): a preplanned interim analysis of a phase 3, non-inferiority, randomised trial. *The Lancet Oncology*. 2025;26(1):37-50. doi:10.1016/S1470-2045(24)00661-2

5. Dumas E, Jochum F, Coussy F, et al. Explaining the Relationships Between Age, Endocrine Therapy Persistence, and Risk of Recurrence in Hormone Receptor–Positive Early Breast Cancer: A Nationwide Cohort Study. *JCO*. 2025;0(0):JCO.24.01131. doi:10.1200/JCO.24.01131

6. Dumas E, Laot L, Coussy F, et al. The French Early Breast Cancer Cohort (FRESH): A Resource for Breast Cancer Research and Evaluations of Oncology Practices Based on the French National Healthcare System Database (SNDS). *Cancers*. 2022;14(11):2671. doi:10.3390/cancers14112671

7. Rey G, Jougla E, Fouillet A, Hémon D. Ecological association between a deprivation index and mortality in France over the period 1997 - 2001: variations with spatial scale, degree of urbanicity, age, gender and cause of death. *BMC Public Health*. 2009;9:33. doi:10.1186/1471-2458-9-33

8. Berger ML, Mamdani M, Atkins D, Johnson ML. Good Research Practices for Comparative Effectiveness Research: Defining, Reporting and Interpreting Nonrandomized Studies of Treatment Effects Using Secondary Data Sources: The ISPOR Good Research Practices for Retrospective Database Analysis Task Force Report—Part I. *Value in Health*. 2009;12(8):1044-1052. doi:10.1111/j.1524-4733.2009.00600.x

© 2025 Dumas E et al. *JAMA Network Open*
