# Supplementary material for: Outcomes of Anastrozole, Letrozole, and Exemestane in Patients With Postmenopausal Breast Cancer
Source: JAMA Netw Open. 2025 Dec 26;8(12):e2550842. doi: 10.1001/jamanetworkopen.2025.50842 (PMC12743288; doi:10.1001/jamanetworkopen.2025.50842)
Supplement: Supplement 2. — Data Sharing Statement [file jamanetwopen-e2550842-s002.pdf]

# Data Sharing Statement

Dumas. Anastrozole, Letrozole, and Exemestane Effectiveness in Patients With Postmenopausal Breast Cancer. *JAMA Netw Open*. Published December 26, 2025. doi:10.1001/jamanetworkopen.2025.50842

**Data**
**Data available:** No

**Additional Information**
**Explanation for why data not available:** According to data protection and French regulation, the authors cannot publicly release the data from the SNDS. However, any person or structure, public or private, for-profit or nonprofit, is able to access SNDS data upon authorization from the French Data Protection Office (CNIL, Commission Nationale de l'Informatique et des Libertés) to carry out a study or an evaluation of public interest.
